# Functional divergence and structural changes of Class IV histone deacetylases (HDACs) across the tree of life

**DOI:** 10.1093/molbev/msag150

**Published:** 2026-06-17

**Authors:** Zora Nováková, Pavla Bartošová-Sojková, Júlia Kudláčová, Fady Baselious, Zsófia Kutilová, Pavlína Jaklová, Marat Meleshin, Lucia Motlová, Andrea Schenkmayerova, Vladimír Vrkoslav, Štěpán Strnad, Natan Horáček, Ansgar Gruber, Petr Žáček, Sebastian Kroll, Barbora Havlínová, Markéta Ondráková, Růžena Tučková, Tereza Krunclová, Josef Cvačka, Miroslav Oborník, Mike Schutkowski, Wolfgang Sippl, Cyril Bařinka

**Affiliations:** Institute of Biotechnology of the Czech Academy of Sciences, BIOCEV, Vestec, Czech Republic; Biology Centre of the Czech Academy of Sciences, Institute of Parasitology, Ceske Budejovice, Czech Republic; Institute of Biotechnology of the Czech Academy of Sciences, BIOCEV, Vestec, Czech Republic; Department of Medicinal Chemistry, Institute of Pharmacy, Martin-Luther-University of Halle-Wittenberg, Halle (Saale), Germany; Institute of Biotechnology of the Czech Academy of Sciences, BIOCEV, Vestec, Czech Republic; Institute of Biotechnology of the Czech Academy of Sciences, BIOCEV, Vestec, Czech Republic; Charles Tanford Protein Center, Department of Enzymology, Institute of Biochemistry and Biotechnology, Martin-Luther-University of Halle-Wittenberg, Halle (Saale), Germany; Institute of Biotechnology of the Czech Academy of Sciences, BIOCEV, Vestec, Czech Republic; Institute of Biotechnology of the Czech Academy of Sciences, BIOCEV, Vestec, Czech Republic; Institute of Organic Chemistry and Biochemistry of the Czech Academy of Sciences, Prague, Czech Republic; Institute of Organic Chemistry and Biochemistry of the Czech Academy of Sciences, Prague, Czech Republic; Institute of Organic Chemistry and Biochemistry of the Czech Academy of Sciences, Prague, Czech Republic; Biology Centre of the Czech Academy of Sciences, Institute of Parasitology, Ceske Budejovice, Czech Republic; Faculty of Science, University of South Bohemia, Ceske Budejovice, Czech Republic; OMICS Mass Spectrometry Core Facility, Biology Department, Faculty of Science, Charles University, BIOCEV, Vestec, Czech Republic; Institute of Biotechnology of the Czech Academy of Sciences, BIOCEV, Vestec, Czech Republic; Institute of Biotechnology of the Czech Academy of Sciences, BIOCEV, Vestec, Czech Republic; Institute of Biotechnology of the Czech Academy of Sciences, BIOCEV, Vestec, Czech Republic; Institute of Biotechnology of the Czech Academy of Sciences, BIOCEV, Vestec, Czech Republic; Institute of Biotechnology of the Czech Academy of Sciences, BIOCEV, Vestec, Czech Republic; Institute of Organic Chemistry and Biochemistry of the Czech Academy of Sciences, Prague, Czech Republic; Biology Centre of the Czech Academy of Sciences, Institute of Parasitology, Ceske Budejovice, Czech Republic; Faculty of Science, University of South Bohemia, Ceske Budejovice, Czech Republic; Charles Tanford Protein Center, Department of Enzymology, Institute of Biochemistry and Biotechnology, Martin-Luther-University of Halle-Wittenberg, Halle (Saale), Germany; Department of Medicinal Chemistry, Institute of Pharmacy, Martin-Luther-University of Halle-Wittenberg, Halle (Saale), Germany; Institute of Biotechnology of the Czech Academy of Sciences, BIOCEV, Vestec, Czech Republic

**Keywords:** histone deacetylase 11, fatty acid deacylase activity, substrate profiling, structural selectivity filter, evolutionary divergence, phylogenetic analysis

## Abstract

Class IV histone deacetylases (HDACs) are the least understood branch of the classical zinc-dependent HDAC family with HDAC11 standing out as the sole member of Class IV HDACs. Using a broad phylogenetic dataset spanning bacteria, archaea, and eukaryotes, we identified two deeply conserved HDAC11 lineages, clades A and B, that differ in evolutionary origin, predicted subcellular localization, and enzymatic properties. Clade A is enriched in phototrophic eukaryotes and targeted to mitochondria or plastids, whereas clade B predominates in heterotrophs and localizes mainly to the cytoplasm or nucleus. High-resolution crystal structures of selected representatives from each clade revealed a conserved catalytic core but distinct structural features—including electrostatic surface profiles, loop architectures, and foot pocket geometries—that clearly separate the two lineages and act as sequential “selectivity filters” shaping substrate specificity. Biochemical assays show robust long-chain fatty acid deacylase activity in clade B enzymes, but no detectable activity for any of clade A representatives against peptide substrates, suggesting adaptation to alternative, nonpeptidic targets. Together, these findings define a revised evolutionary framework for HDAC11 and provide structural and functional insights into the diversification of this ancient enzyme family.

## Graphical Abstract

**Graphical Abstract:**
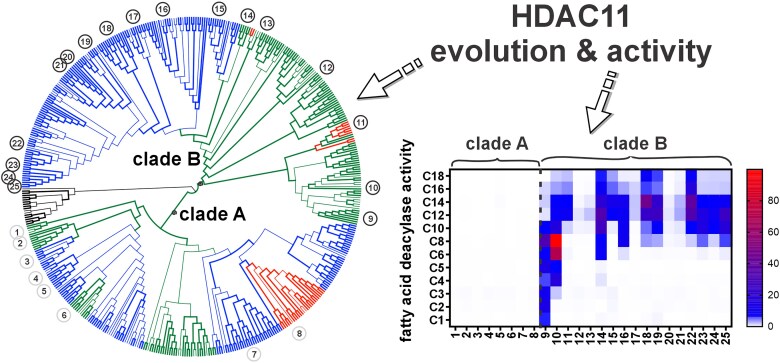

## Introduction

Histone deacetylases (HDACs) are evolutionarily conserved enzymes found across all domains of life, emphasizing their fundamental roles in cellular regulation. In eukaryotes, classical zinc-dependent HDACs are categorized into three major classes, Class I, II, and IV, based on sequence similarity, domain architecture, and enzymatic properties, each reflecting distinct evolutionary pathways and biological functions (Milazzo et al. 2020). Class III HDACs, or sirtuins, are mechanistically distinct, utilizing NAD^+^ as a cofactor and forming a separate structural lineage (Gregoretti et al. 2004). Intriguingly, homologs of classical HDACs have been identified in bacteria, suggesting that these enzymes predate the emergence of histones and initially targeted nonhistone substrates, including small molecules and polyamines (Leipe and Landsman 1997; Hai et al. 2017; Graf et al. 2024). This ancestral diversity underlines the broader functional repertoire of HDACs beyond chromatin regulation.

Among classical HDACs, HDAC11 stands out as the only member of Class IV and exhibits unique biochemical characteristics. Despite its structural similarity to Class I and II enzymes, human HDAC11 lacks canonical deacetylase activity and instead preferentially removes long-chain fatty acyl groups from lysine residues (Kutil et al. 2018; Moreno-Yruela et al. 2018; Cao et al. 2019). This defatty-acylase activity is modulated by metabolic signals, such as activation by myristoyl-CoA and inhibition by free fatty acids, implicating HDAC11 in lipid signaling and metabolic regulation (Kutil et al. 2019). In murine models, HDAC11 deficiency leads to diverse phenotypes, including enhanced thermogenesis, improved insulin sensitivity, and protection against high-fat diet-induced obesity (Seto and Yoshida 2014; McCullough and Marmorstein 2016; Sun et al. 2018). HDAC11 also regulates immune functions, influencing IL-10 expression, antigen presentation, and regulatory T-cell activity, pointing to broader roles in immunometabolic homeostasis (Villagra et al. 2009; Woods et al. 2017; Nunez-Alvarez and Suelves 2022). Notably, while HDAC11 knockout mice are viable, RNAi-mediated depletion of HDAC11 in the beetle *Tribolium castaneum* results in lethality, suggesting divergent functional constraints across taxa (George and Palli 2020).

Despite increasing interest in HDAC11, its evolutionary origins and structural diversification remain poorly understood. A foundational study by Ledent and Vervoort (2006) proposed two divergent lineages within Class IV HDACs: one consisting solely of eukaryotic homologs and another containing both bacterial and eukaryotic sequences, hinting at potential ancient horizontal gene transfer, a phenomenon well-documented in gene evolution (Ochman et al. 2000; Boto 2014). Recent comprehensive bacterial surveys have identified Class IV HDACs as a phylogenetically distinct clade of zinc-dependent HDACs, encompassing a diverse array of classical deacylases from bacteria, mammals, and plants (Graf et al. 2024). However, HDAC11 remains insufficiently characterized at the structural level, and its biochemical specialization remains unclear.

In this study, we integrate phylogenetics, enzymology, and structural biology to resolve the evolutionary and functional diversification of HDAC11. Our analysis reveals two deep-branching clades, A and B, distinguished by their substrate preferences, structural features, and subcellular localizations, providing new insights into the functional divergence of this atypical HDAC.

## Results

### Phylogenetic landscape and divergence of Class IV HDACs

**Figure 1:**
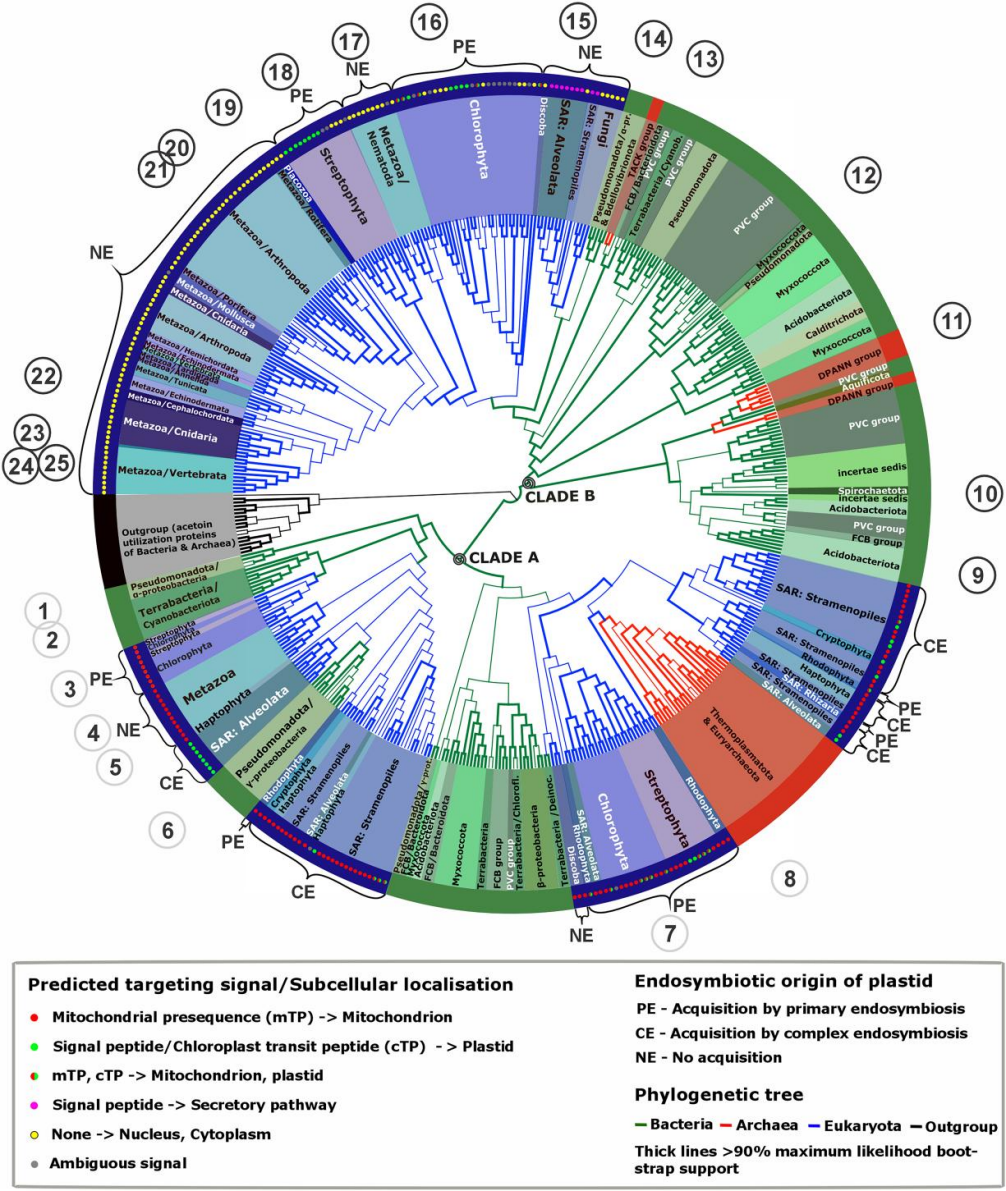
Circle-formatted maximum likelihood phylogenetic tree of 480 Class IV HDACs, with predictions of targeting presequences, associated subcellular localization, and types of plastid endosymbiosis mapped in the outer circle for each HDAC sequence. The tree branches are color-coded according to the main domains of life: the Eukaryota, Bacteria, and Archaea. The thick lines indicate >90% bootstrap support in the analysis. Detailed information about predictions is provided in Table S1. The original tree is available in Figure S3. Positions of species from clade A and B selected for biochemical analysis (Table 1) are shown by numbers in gray and black circles around the tree, respectively.

To place HDAC11 within the broader context of Class IV HDAC evolution, we assembled a phylogenetically diverse dataset of bacterial, archaeal, and eukaryotic sequences and reconstructed their relationships using the maximum likelihood (ML) method. Our comprehensive phylogenetic analysis of classical HDACs uncovered three well-supported clades corresponding to Classes I, II, and IV (Figures S1, S2). Within Class IV, we identified two robustly supported subclades, A and B, each defined by conserved motifs and distinct evolutionary signatures (Fig. 1; Figures S3, S4). While all HDAC classes retain the four canonical motifs within the catalytic domain, specific residues within these motifs diverge across classes and even between clades A and B of Class IV, indicating functional diversification (Figure S4).

Data mining revealed that Class IV HDACs are broadly distributed across eukaryotes and bacteria, but are rare in archaea. Within eukaryotes, Class IV HDACs appear in various taxa, including fungi and apicomplexans, groups previously reported to lack the enzyme of this class (Ledent and Vervoort 2006). However, the limited number of homologs, restricted to a few taxa, and their long branches in our phylogenetic tree of Class IV HDACs suggest sparse conservation and rapid divergence in the mentioned eukaryotic groups (Figure S3).

Clades A and B contain homologs from bacteria, archaea, and eukaryotes (Fig. 1; Figure S3), but do not completely follow the expected tree topology of the species, suggesting events such as gene duplications, horizontal gene transfers, or gradual gene loss. Clade A contains α-proteobacterial HDACs at its root, while clade B originates from a diverse collection of bacterial lineages (Acidobacteria, PVC group, FCB group, Spirochaetota) and DPANN Archaea. Strikingly, most bacterial diversity occurs in both clades, while archaeal representatives are rare and typically nested within bacterial or eukaryotic lineages in the A and B clades, respectively, suggesting either contamination or gene transfer from bacteria to archaea. Eukaryotes are also distributed across both clades. However, most species, especially most metazoans, harbor only one enzyme of the Class IV HDAC, which is present in one of the two clades. The exceptions include streptophytes (land plants), chlorophytes (green algae), and some metazoans (eg *Gasterosteus aculeatus aculeatus*, *Locusta migratoria*, and *Nematostella vectensis*), which code for multiple paralogs from both clades (Tables S1, S2). Overall, our analyses resolved two deeply branching HDAC11 lineages, hereafter referred to as clades A and B, that are conserved across domains of life and form the basis for subsequent structural and functional comparisons.

### Predicted subcellular localization of HDAC11s

To explore whether clade association correlates with distinct cellular roles, we predicted the subcellular localization of all HDAC11 homologs in our dataset using multiple independent prediction tools. In clade A, HDACs predominantly contain mitochondrial or chloroplast transit peptides, and many plastid-targeted HDACs in complex algae have bipartite targeting presequences consisting of an endoplasmic reticulum (ER) signal peptide and a chloroplast transit peptide. Ambiguous dual-targeting signals for both mitochondria and plastids have been identified in several diatoms, green and red algae, and early branching land plants. Experimental observations agree with our predictions for enzyme localization in *Toxoplasma gondii* and related parasites (*Besnoitia*, *Cystoisospora*, *Neospora*, *Plasmodium*).

In contrast, clade B eukaryotic HDACs are mostly predicted to localize in the cytoplasm or nucleus, with some notable exceptions. Several members of the clade, particularly chlorophytes and angiosperms (flowering land plants), exhibit plastidial or dual plastidial and mitochondrial localization, while many land plants (such as liverworts, mosses, and basal angiosperms such as *Amborella*) exhibit cytoplasmic/nuclear localization or ambiguous targeting signals to multiple organelles. In addition, clade B HDACs in ciliates contain enzymes with transmembrane domains (signal anchors) that facilitate co-translational targeting to the endoplasmic reticulum (Fig. 1, Table S1).

When multiple paralogs exist within a single species, they typically divide into A and B clades with different localization signals, underscoring possible functional diversification (Table S2). This distribution suggests that intracellular localization, determined by N-terminal targeting signals, has co-evolved with structural divergence to support specialized roles of HDAC11 in different cellular compartments.

**Figure 2:**
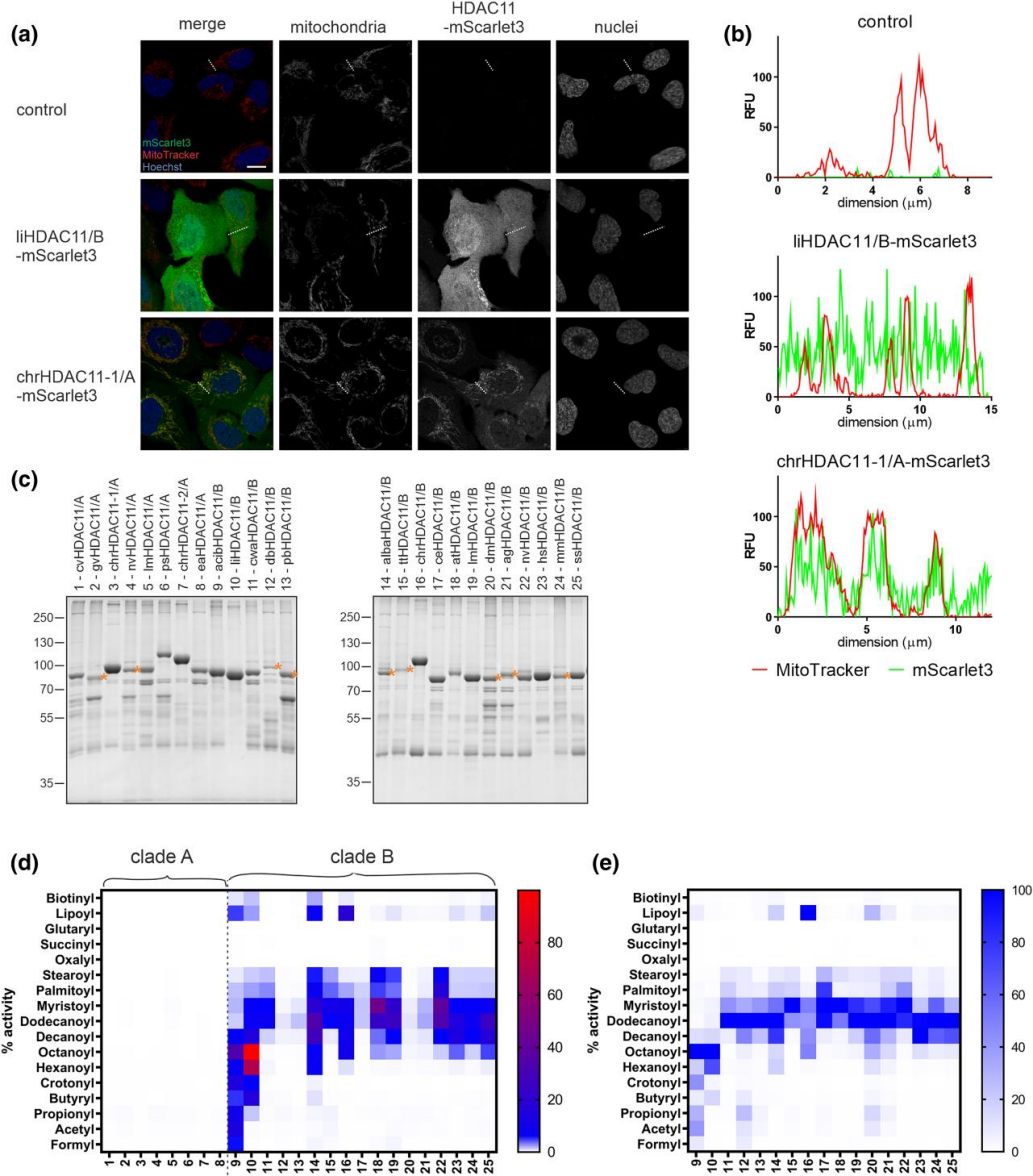
Expression, localization, and activity profiling of HDAC11 homologs. (a) Subcellular localization of representative HDAC11 homologs from clade A (chrHDAC11-1/A; native sequence containing a mitochondrial transit peptide) and clade B (liHDAC11/B; no transit peptide in native sequence). Live-cell imaging of HDAC11-mScarlet3 fusion constructs was performed by confocal microscopy. HDAC11 constructs were imaged via signal of fused mScarlet3 green, while mitochondria and nuclei were visualized by MitoTracker Deep Red FM (red) and Hoechst 33258 (blue), respectively. The clade B representative showed diffuse cytoplasmic and nuclear signal, while the clade A homolog localized preferentially to mitochondria. Bar: 20 µm. (b) Intensity profiles along the white dashed line in (a) show substantial colocalization of the mitochondrial signal (red) with chrHDAC11-1/A (green) but not with liHDAC11/B. (c) SDS-PAGE analysis of (semi)purified HDAC11 homologs expressed in HEK293T cells. Proteins were affinity-purified using an N-terminal Strep-FLAG-HALO tag and resolved on a 12% polyacrylamide gel. Variability in expression level and purity reflects intrinsic differences in stability and purification yield among homologs. In complex/low-purity samples, HDAC11 proteins are marked by orange asterisks. (d) Heatmap of deacylase activity of HDAC11 homologs against a panel of acylated peptide substrates. Purified proteins were incubated with a synthetic peptide library, and enzymatic activity was quantified by HPLC. Activity is shown as a double gradient heatmap normalized to the highest value in the whole protein set. Values of the heatmap together with the raw data are shown in Table S3. Clade B HDAC11s generally preferred medium- to long-chain fatty acyl substrates, whereas homologs at the base of the clade showed preference for shorter chains. No deacylase activity was detected for clade A HDAC11s. (e) Heatmap of HDAC11 homologs included in clade B. Activity of each homolog is normalized individually, with the value for the best substrate set to 100%.

To experimentally assess these predictions, we overexpressed a representative clade A enzyme containing a native mitochondrial transit peptide, chrHDAC11-1/A, and a clade B representative lacking an apparent signal peptide, liHDAC11/B, in human U-2 OS cell line. Both proteins were fused at the C-terminus to mScarlet3 and expressed from a PiggyBac transposon vector under cumate control (Figure S5), enabling low-level expression compatible with organelle-directed targeting. Confocal microscopy combined with vital staining of nuclei and mitochondria using Hoechst 33258 and MitoTracker Deep Red FM, respectively, revealed diffuse cytoplasmic and nuclear localization of liHDAC11/B-mScarlet3, whereas chrHDAC11-1/A-mScarlet3 localized predominantly to mitochondria (Fig. 2a, b). Thus, the experimental data are consistent with the localization patterns predicted for the two clades.

### Phylogenetic clustering of Class IV HDACs and association with trophic strategies and plastid evolution

We next examined whether clade distribution correlates with organismal traits, focusing on trophic strategies and the evolutionary history of plastids. Clade A is dominated by phototrophic eukaryotes, ie species with either primary plastids (eg glaucophytes, red algae, green algae, and land plants) or complex plastids acquired through secondary or higher-order endosymbioses (eg diatoms, cryptophytes, and alveolates). Eukaryotic heterotrophs are underrepresented in clade A, and they usually have only a single HDAC11 homolog from clade B. There are a few exceptions: Certain metazoans (eg cnidarians, echinoderms, mollusks, annelids, arthropods, and early branching vertebrates) and amoebae (*Naegleria* spp.) encode clade A HDACs that are predicted to be localized in mitochondria. Importantly, species of the aforementioned metazoan groups also possess clade B paralogs, revealing a rare but functionally distinct conservation pattern (Table S1).

In vertebrates, the representation of dual clades is restricted to the basal groups. Teleost fishes such as *Danio rerio*, *G. a. aculeatus*, *Oryzias latipes*, and *Pimephales promelas* as well as the coelacanth *Latimeria chalumnae* possess both A and B paralogs (Table S1), which were previously suggested to have arisen by gene duplication in certain bony fish clades (Gregoretti et al. 2004). The teleost *Takifugu rubripes* encodes only a clade A HDAC (Table S1), and our broader survey of other tetraodontiform fish genomes confirmed the apparent absence of clade B homologs in this fish order (not shown). Cartilaginous fish and higher vertebrates, including amphibians, reptiles, birds, and mammals, have only clade B paralogs (Table S1). Importantly, our analysis of genomic context rules out bacterial contamination for clade A HDACs in metazoan genomes, as these genes are always flanked by eukaryote-specific loci (not shown).

Overall, these patterns suggest that clade A HDACs are functionally associated with organelles of endosymbiotic origin, whereas clade B homologs predominate, with some exceptions, in the nucleus and cytosol of heterotrophs. The rarity of clade A HDACs in nonphotosynthetic organisms could be due to ancient gene duplications followed by clade-specific conservation, loss, or compartmental specialization.

### Selection, expression, and purification of HDAC11 homologs

To capture the evolutionary and functional diversity of Class IV HDACs, we selected 25 representative homologs from diverse taxonomic groups for biochemical and structural analyses (Fig. 1, Table 1). Selection criteria included (i) representation from key evolutionary lineages associated with the endosymbiotic origin of organelles (eg cyanobacteria for plastids, α-proteobacteria for mitochondria); (ii) species with multiple Class IV HDAC paralogs (eg *Chlamydomonas reinhardtii*, *N. vectensis*, *L. migratoria*); (iii) broad taxonomic sampling within Metazoa (eg cnidarians, arthropods, nematodes, and vertebrates); (iv) sequences located near the base of clade A or B to infer ancestral substrate preferences; and (v) unique substitutions within conserved sequence/structural motifs. Genes encoding selected HDAC11 homologs were codon-optimized, synthesized, and cloned into the mammalian expression vector pMM322 as fusion proteins carrying an N-terminal Twin-Strep-FLAG-HALO tag (Skultetyova et al. 2017) (Figure S5). Next, individual proteins were expressed in HEK293T cells and purified via Strep-Tactin affinity chromatography (Fig. 2c and Figure S6). The resulting panel of purified proteins provided a robust experimental basis for biochemical characterization and structural determination.

**Table 1:** List of representative HDAC11 homologs.

Number	Species	Abbreviation	Accession no.
	**Clade A**		
1	*Caulobacter vibrioides*	cvHDAC11/A	WP_010921475.1
2	*Gloeobacter violaceus*	gvHDAC11/A	BAC89560.1
3	*Chlamydomonas reinhardtii*	chrHDAC11-1/A	XP_042917102.1
4	*Nematostella vectensis*	nvHDAC11/A	XP_001640343.3
5	*Locusta migratoria*	lmHDAC11/A	GETS01106794.1
6	*Pseudomonas syringae*	psHDAC11/A	KPW24014.1
7	*Chlamydomonas reinhardtii*	chrHDAC11-2/A	XP_042921739.1
8	Euryarchaeota archaeon	eaHDAC11/A	GIR80856.1
	**Clade B**		
9	*Acidobacteriia bacterium*	acibHDAC11/B	SPE23023.1; WP_105313729
10	*Leptospira interrogans*	liHDAC11/B	NP_713103.1,_WP_000410917.1
11	Candidatus Woesearchaeota archaeon	cwaHDAC11/B	MBS3118946.1
12	*Deltaproteobacteria bacterium*	dbHDAC11/B	HCH63001
13	*Pseudanabaena biceps*	pbHDAC11/B	WP_009625954
14	*Alphaproteobacteria bacterium*	albaHDAC11/B	TNE32314
15	*Tetrahymena thermophila*	ttHDAC11/B	XP_001011943.2
16	*Chlamydomonas reinhardtii*	chrHDAC11/B	XP_042925771.1
17	*Caenorhabditis elegans*	ceHDAC11/B	NP_505699.3
18	*Arabidopsis thaliana*	atHDAC11/B	NP_568480.2
19	*Locusta migratoria*	lmHDAC11/B	GEZB01017848.1
20	*Drosophila melanogaster*	dmHDAC11/B	NP_001247296.1
21	*Anopheles gambiae*	agHDAC11/B	XP_321350.6
22	*Nematostella vectensis*	nvHDAC11/B	XP_001636500.2
23	*Homo sapiens*	hsHDAC11/B	NP_079103.2
24	*Mus musculus*	mmHDAC11/B	NP_659168.1
25	*Sus scrofa*	ssHDAC11/B	XP_020925043.1

### Biochemical characterization of HDAC11 substrate specificity

We evaluated the substrate preferences of clade A and clade B enzymes using a panel of 17 synthetic fluorogenic peptidic substrates harboring different acyl chains at the central lysine (Kutil et al. 2018; Figure S7). Each enzyme (at concentrations estimated via SDS-PAGE densitometry) was incubated with 10 µM of a tested substrate at 37 °C for 30 min, and reaction products were analyzed by HPLC (Figure S8).

All clade B HDAC11 enzymes displayed clear deacylation activity, with a general preference for peptides modified with medium- to long-chain fatty acids (C10−C18; Fig. 2d, e, and Table S3). Among these, dodecanoyl and myristoyl substrates were typically the most efficiently hydrolyzed. In contrast, negatively charged acyl groups such as oxalyl, succinyl, and glutaryl were not deacylated under the tested conditions. Notably, homologs situated at the base of the HDAC11 evolutionary tree, including those from *Acidobacteriia*, *Leptospira interrogans*, and *Deltaproteobacteria*, accept and prefer shorter acyl chains (acetyl to octanoyl), suggesting that early Class IV HDACs may have initially evolved as short-chain deacylases, with later specialization toward long-chain specificity.

Unexpectedly, none of the clade A homologs tested exhibited measurable deacylase activity against any of the substrates in the assay panel (Fig. 2d), highlighting a potential loss or divergence of enzymatic function in this clade. These results underscore a functional divergence between HDAC11 clades. Clade B enzymes consistently retain long-chain fatty acid deacylase activity within the peptide context, while Clade A members appear catalytically inactive against peptide-based substrates under our assay conditions, pointing to possible adaptation to alternative substrates.

### Structural characterization of the clade A and B representatives—conservation of the catalytic site

**Figure 3:**
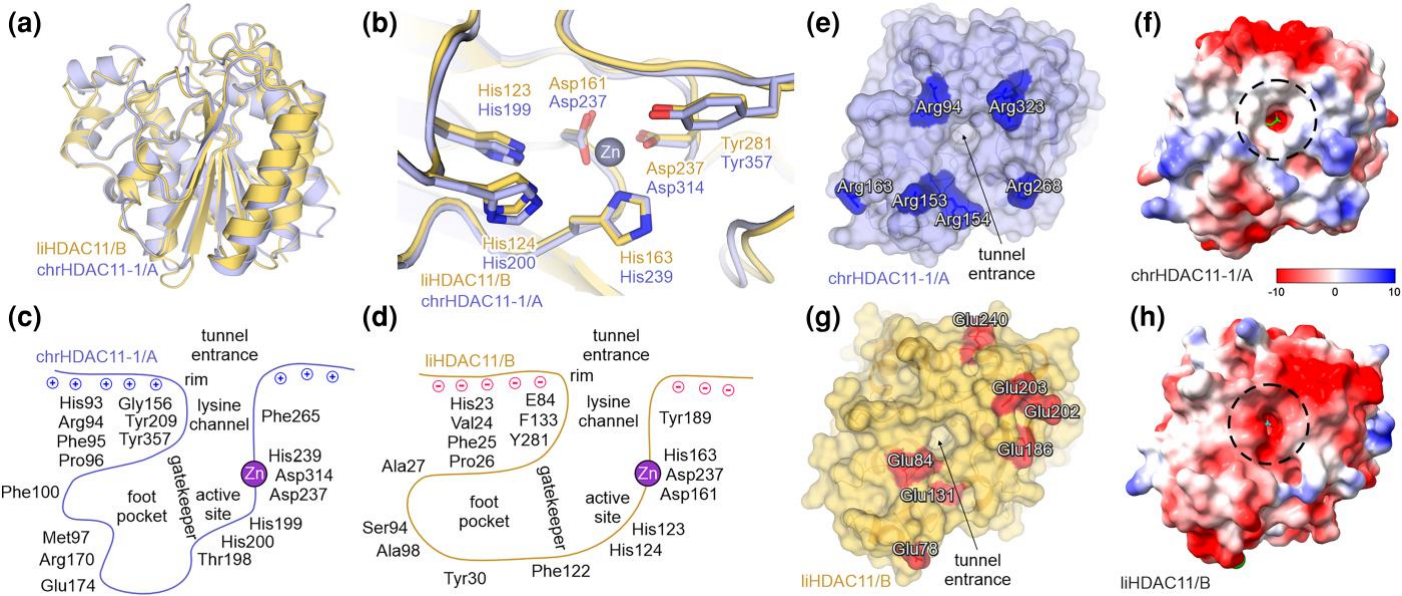
Structural characterization of clade A and B HDAC11 representatives. (a) Crystal structure of chrHDAC11-1/A superimposed to liHDAC11/B in cartoon representation. Both experimental structures adopt the identical canonical α/β-hydrolase (arginase/deacetylase) fold. (b) Details of the HDAC11 active site (superposition of chrHDAC11-1/A and liHDAC11/B). The Zn-coordinating (Asp161/237, Asp237/314, Tyr281/357) and catalytic residues (His123/199, His124/200) are shown as sticks. (c) 2D scheme representing the internal pocket of chrHDAC11-1/A and (d) liHDAC11/B. (e, f) The surface representations of chrHDAC11-1/A with arginine residues marked in dark blue (e) or colored by electrostatic potential (f). (g, h) The surface representations of liHDAC11/B with glutamate residues marked in red (g) or colored by electrostatic potential (h). Electronegative regions are shown in red, electropositive regions in blue, and neutral regions in white (bar: -10 to +10 kcal mol^-1^ e-1). The chrHDAC11-1/A tunnel entrance displays neutral to positive electrostatic potential, while the liHDAC11/B tunnel entrance exhibits negative electrostatic potential (tunnel entrance encircled by a dashed line).

To identify structural features underlying the divergent activities of clade A and B enzymes, we determined crystal structures of one representative from each clade—*L. interrogans* (clade B; liHDAC11/B) and *C. reinhardtii* (clade A; chrHDAC11-1/A), resolved at 1.51 and 1.06 Å resolution limits, respectively (Tables S4 and S5). Additionally, we generated *in silico* comparative models for the remaining HDAC11 homologs for structural analyses of conserved motifs to complement the sequence phylogeny analyses (Figures S9 to S33). Both experimental structures adopt the identical canonical α/β-hydrolase (arginase/deacetylase) fold with a core RMSD of ∼1.3 Å (SSM superposition; Fig. 3a), which is also preserved in homology models. The catalytic machinery, including the Zn^2+^ coordination sphere and residues essential for catalysis, is fully conserved in both clades (Fig. 3b and Figure S34; Table S4). Additionally, in both experimental structures, a K^+^ ion is positioned ∼7 Å from the zinc center (Figure S34c, d) in a location consistent with a proposed catalytic role (Gantt et al. 2010). Finally, two aromatic residues, Phe133/Tyr189, and Tyr209/Phe265 in liHDAC11/B and chrHDAC11-1/A, respectively, equivalent to Phe152 and Tyr209 in human HDAC11 (NCBI accession no. NP_079103.2) line the substrate-binding channel, forming a conserved hydrophobic surface for acyl-lysine recognition (Weerasinghe et al. 2008) (Fig. 3c, d, Figure S35). Given this high degree of active-site conservation, the lack of detectable deacylase activity in clade A enzymes must arise from structural differences outside the immediate catalytic core.

### Surface electrostatic potential as a potential first “selectivity filter”

Although the overall fold, substrate-binding tunnel, and catalytic residues are conserved between clade A and clade B HDAC11s, the composition of residues at the active-site entrance, rim, and foot pocket differs markedly (Fig. 3c, d). In chrHD11-1/A (clade A), multiple arginine residues (Arg94, Arg153, Arg154, Arg163, Arg268, Arg323) cluster near the tunnel entrance (Fig. 3e), creating a predominantly electropositive-to-neutral surface (Fig. 3f). In contrast, liHD11/B (clade B) features several surface-exposed glutamate residues (Glu78, Glu84, Glu131, Glu186, Glu202, Glu203, and Glu240) at the tunnel entrance face (Fig. 3g), generating a more electronegative potential (Fig. 3h). This trend is conserved across structural models from each clade, with clade A enzymes generally showing electropositive-to-neutral entrances and clade B enzymes displaying more negative electrostatic potential (Figures S36 and S37). Such differences may act as a first “selectivity filter,” where electrostatic complementarity influences substrate affinity and productive enzyme/substrate complex formation. In contrast, hydrophobicity profiles of the tunnel entrances are similar in both structures described above, with no major differences in hydrophobic/hydrophilic region distribution (Figure S38).

### Rim residues as a second “selectivity filter”

**Figure 4:**
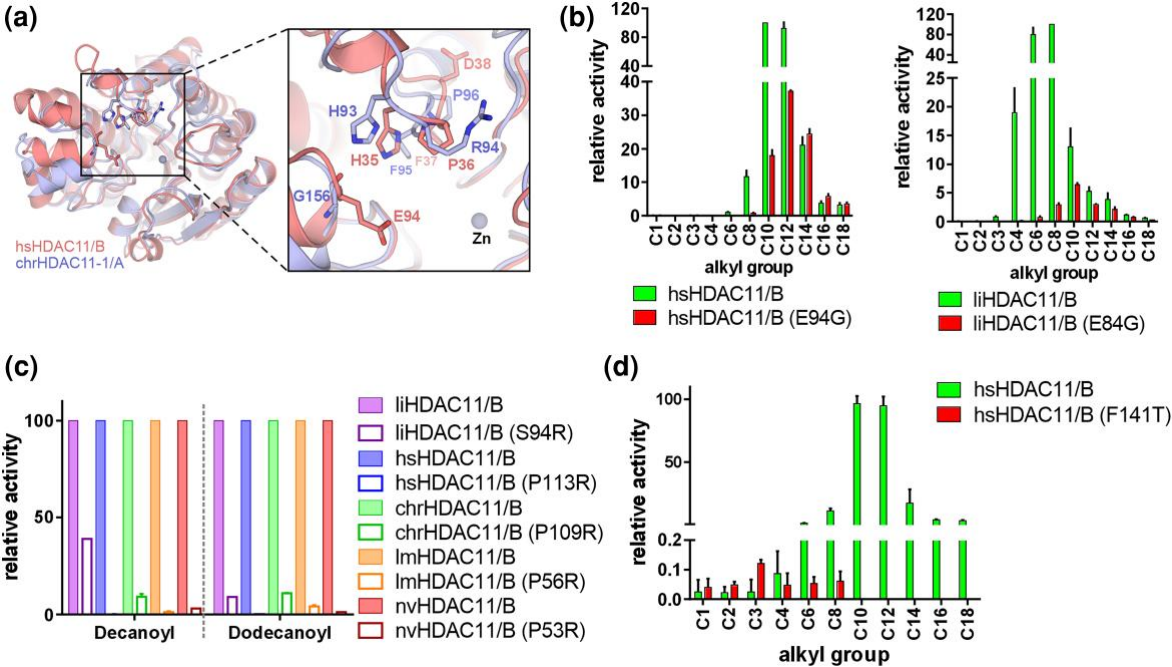
Identification of motifs critical for Class B enzymatic activity. (a) Superposition of chrHDAC11/A (blue) and hsHDAC11/B (red) structures with the loop motifs highlighted (shown as sticks). Clade B enzymes feature the HPFD motif and glutamate at position 94 (human numbering), while proline in the HPFD motif is replaced by arginine, and Glu94 with glycine in Class A proteins. (b) Comparison of enzymatic activities of wild-type and mutant hsHDAC11/B (E94G) and liHDAC11/B (E84G) enzymes. The highest activity in each profile was arbitrarily set to 100%, *n* = 3. While the mutations lead to general decrease in enzymatic activity, the decrease is not uniform across the substrate panel pointing toward cross talk between the rim residues and the foot pocket harboring acyl groups. (c) Comparison of enzymatic activities of wild-type and mutant (the end of the foot pocket) HDAC11s. The activities of wild-type enzymes were arbitrarily set to 100%, *n* = 3. (d) The substrate specificity of wild-type hsHDAC11/B and its F141T mutant where the gatekeeper phenylalanine residue typical to clade B was mutated to threonine (the clade A signature).

The rim and the walls of the substrate-binding tunnel are formed by several loops, most notably loop 1 (L1; residues 35-40, hsHDAC11/B numbering), loop 2 (L2; residues 86-100), and loop 3 (L3; residues 137-156) that have been implicated in substrate processing and inhibitor binding in other classical HDACs (Vannini et al. 2007; Hai and Christianson 2016; Osko et al. 2020; Shukla et al. 2021). In clade B, L1 (between β2 and α2) contains the consensus H_35_P_36_F_37_D_38_ motif (Figure S39) forming one of the gates to the active-site tunnel where Pro36 contributes to loop rigidity and influences the positioning of Phe152 (L3), a residue involved in both substrate and inhibitor binding in HDAC8 and HDAC11 (Baselious et al. 2023). In clade A, Pro36 of the HPFD motif is replaced by a conserved arginine, altering loop size and flexibility (Fig. 4a; Table S4).

Another signature residue of the clade B is Glu94 (in hsHDAC11/B) located at L2 which is replaced by glycine in the clade A enzymes (Fig. 4a and Figure S39; Table S4). In human HDAC8, the corresponding Asp101 is implicated in positioning substrates by directly interacting with the amide nitrogen of the peptidic backbone of a substrate (Vannini et al. 2007). The presence of the conserved glutamate in clade B HDACs thus implies that these enzymes favor peptides/proteins as substrates, while its absence in clade A suggests specification toward the yet-to-be identified nonpeptidic/nonprotein small-molecule substrates, such as carbohydrates, small molecules, antibiotics, and polyamines (Blair et al. 2005; Hai et al. 2017; Burger and Chory 2018). The importance of Glu94 for defatty-acid activity of clade B representatives is documented by site-directed mutagenesis on hsHDAC11/B (E94G) and liHDAC11/B (E84G), where the mutations mostly reduced enzyme activity toward acylated substrates, reaching up to 35-fold reduction for octanoylated peptide in the case of liHDAC11/B (E84G). Interestingly, the activity decrease was not uniform across the substrate panel, with more pronounced effect observed for shorter acyl groups (C1 to C8), while the substrates harboring longer acyl groups (C10 to C18) were less affected, with the trend even reversed for hsHDAC11/B against C14 to C18 harboring peptides (Fig. 4b). These findings point toward the cross talk between individual “selectivity filters” in HDAC11 enzymes. Together, the HPFD motif composition and the identity of residue 94 form the second “selectivity filter” that likely modulates the clade A vs clade B substrate specificity.

### Foot pocket as an acyl-chain “selectivity filter”

HDAC11’s unique preference for long fatty acyl substrates (Kutil et al. 2018; Moreno-Yruela et al. 2018; Cao et al. 2019) is reflected in the architecture of the foot pocket, which also differs between chrHDAC11-1/A and liHDAC11/B (and clade A and B enzymes *in extenso*) (Figure S35). In liHDAC11/B (clade B), Phe122 (corresponding to Phe141 in human HDAC11) serves as a gatekeeper at the pocket base and, together with the side chains of Phe25, Gly132, Phe133, Cys134, Ala278, Gly279, Gly280, and Tyr281, defines the walls of the proximal segment of the pocket. The foot pocket then extends further with a linear architecture being shaped by the side chains of Leu18, Leu20, Phe25, Ala27, Tyr30, Leu85, and Ile91, at its distal part. The bottom of the pocket is terminated by amino acids Ser94, Ala98, and Tyr16 (Fig. 3d, Figure S35). The foot pocket extends to approximately 17 Å, with the total volume of ∼461 Å^3^ (chain A; and 588 A^3^ for chain B), a high hydrophobicity score of 48.4 (46.4 for chain B, calculated by FPocketWeb; Kochnev and Durrant 2022).

In contrast, chrHDAC11-1/A features a shorter bifurcated pocket of a smaller size (301 Å^3^; Fig. 3c, Figure S35). Importantly, the chrHDAC11-1/A pocket is substantially more polar with a hydrophobicity score of 16.3, and its polar nature is underscored by the presence of a network of 10 water molecules filling most of the pocket volume. The pocket is terminated and shortened by the Arg170-Glu174 salt bridge (replacing Ser94 and Ala98 in liHDAC11/B) with interatomic distances of 2.9 and 3.5 Å as well as by substitution of Ala27 with bulky Met97 (Figure S35a). These features, particularly the Arg–Glu pair, are conserved signatures of clade A (Table S4). Site-directed mutagenesis confirmed the functional importance of these substitutions: replacing Ser94 in liHDAC11/B or its structural equivalents in other clade B enzymes, hsHDAC11/B(P113R), chrHDAC11/B(P109R), lmHDAC11/B(P56R), and nvHDAC11/B(P53R), with arginine reduced their enzymatic activity by >95% (Fig. 4c).

Another conspicuous difference is observed at the position of the foot pocket gatekeeper. Clade B typically harbors bulky hydrophobic residues (Phe/Tyr), while clade A features smaller polar residues (Thr/Ser) at the equivalent position (Fig. 3c, d, Table S4). The substitution of Phe141 gatekeeper by threonine (typical of clade A) in several clade B representatives [hsHDAC11/B (F141T), liHDAC11/B (F122T), atHDAC11/B (F199T), nvHDAC11/B (F158T)] reduced deacylase activity to background levels (Fig. 4d; Figure S40). The foot gatekeeper is thus one of the critical signature motifs defining both the catalytic activity and the acyl specificity of clade A vs clade B enzymes (see also below).

### Foot pocket ligands in clade A and B HDAC11s reveal distinct substrate preferences

**Figure 5:**
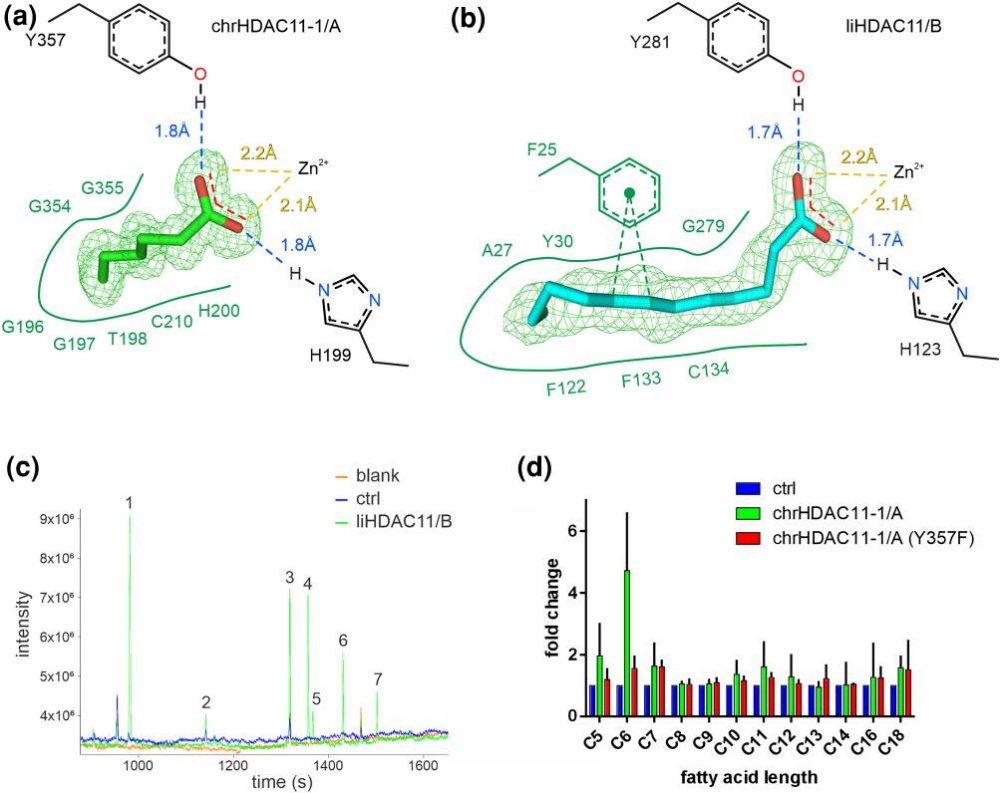
Identification of foot pocket-resident ligands in HDAC11 preparations. Hexanoic acid (a; green sticks) and dodecenoic acid (b; cyan sticks) were modeled in the foot pocket of chrHDAC11-1/A and liHDAC11/B, respectively. *F*o–*F*c maps (green mesh) for ligands are contoured at 2.0 σ. Hydrogen bonds are shown as blue dashed lines, metal-coordinating bonds are yellow dashed lines, and CH-*π* interactions are shown as green dashed lines. The hydrophobic foot pocket is represented as a green line with amino acids shaping the foot pocket and engaged in hydrophobic interactions with the ligand listed (cut off at 4 Å). (c) GC–MS analysis of liHDAC11/B preparation revealed the presence of a mixture of C12 to C18 fatty acids (TIC chromatogram; 1, cis-dodec-5-enoic acid; 2, myristic acid; 3, palmitic acid; 4, palmitoleic acid; 5, hexadec-11-enoic acid; 6, heptadec-7-enoic acid; 7, cis-vaccenic acid), with the cis-dodec-5-enoic acid being the most abundant. The identity of compounds 5 and 6 was not confirmed by identical standards. The double bond position was assigned based on the retention behavior with respect to the known compounds. Blank and control represent vehicle and assay background, respectively. (d) GCxGC–MS analysis identified hexanoic acid in the wild-type chrHDAC11-1/A preparation but not in the Y357F mutant. Data were normalized to the control sample (assay background).

Inspection of the crystal structures revealed electron densities in the foot pockets that were modeled as dodecenoic acid (liHDAC11/B) and hexanoic acid (chrHDAC11-1/A) (Fig. 5a, b). In both cases, the fatty acid carboxylate coordinates the Zn^2+^ ion and forms hydrogen bonds with catalytic histidine and tyrosine residues (liHDAC11/B: H123/Y281; chrHDAC11-1/A: H199/Y357) and are thus positioned in line with their expected placement as products of substrate hydrolysis by the enzymes. The hydrophobic tails occupy the foot pocket running perpendicularly to the entrance tunnel.

As neither of the fatty acids was used in our purification or crystallization protocols, the fatty acids very likely represent products of hydrolysis of “natural” substrates present in *Escherichia coli* used as a host for heterologous expression. To identify exact carboxylic acids in the enzyme preparations, we performed GC–MS and GCxGC–MS analyses. Further, we independently confirmed GC–MS data by an orthogonal approach employing *4-I-AMPP^+^* derivatization followed by UHPLC–MS analysis (see Supplementary material; Narreddula et al. 2019). For the liHDAC11/B, the analyses identified a mixture of C12 to C18 fatty acids, and this finding is consistent with the broad specificity of the enzyme from our biochemical experiments (Fig. 5c, Figure S41a). For chrHDAC11-1/A, only hexanoic acid was detected (Fig. 5d, Figure S41b,c), occupying only the proximal pocket segment, with the remainder of the pocket filled by water molecules. Interestingly, mutation of the active-site Tyr357 to phenylalanine caused the absence of hexanoic acid in protein preparation suggesting fatty acid to be a product of enzymatic activity (Fig. 5d). Despite being well retained and accommodated within the foot pocket, it is surprising that hexanoyl-lysine peptides were not hydrolyzed by chrHDAC11-1/A (and other clade A homologs). It is apparent that the selectivity filters discussed above are not compatible with peptidic substrates used, and these findings warrant further investigation into the identification of clade A natural substrates.

## Discussion

Our phylogenetic analysis demonstrates that Class IV HDACs are divided into two major clades, A and B, each containing representatives from bacteria, archaea, and eukaryotes. This phylogenetic structure differs from an earlier classification that separated Class IV HDACs into a purely eukaryotic group and a mixed bacterial–eukaryotic group (McCullough and Marmorstein 2016), likely reflecting the smaller and less diverse dataset used in that study. Enzymes in clades A and B differ markedly in their predicted subcellular localization. Eukaryotic clade A members are typically targeted to mitochondria or plastids, whereas clade B enzymes occur mainly in heterotrophic organisms and are predicted to localize to the cytoplasm and/or nucleus. Experimental localization data remain scarce. For clade A, experimental data are available only for the Class IV HDAC of the apicomplexan parasite *T. gondii* (Sheiner et al. 2013), which localizes to the rhodophyte-derived, nonphotosynthetic relict plastid (apicoplast) (Lopez-Garcia and Moreira 2019). This organelle hosts essential metabolic pathways, including fatty acid synthesis (FAS), isoprenoid synthesis, and part of heme biosynthesis (Williams et al. 2000). Experimental observations agree with our predictions for enzyme localization in *T. gondii* and related parasites (*Besnoitia*, *Cystoisospora*, *Neospora*). Moreover, we extend experimental evidence by microscopy analysis of the clade A HDAC11 from *C. reinhardtii* which localized to mitochondria in agreement with our predictions. Human HDAC11, a clade B enzyme, predominantly localizes to the nucleus and/or cytoplasm (Gao et al. 2002; Huang et al. 2017; Nunez-Alvarez and Suelves 2022) of distinct cell types, and although mitochondrial localization in skeletal muscle has been reported (Hurtado et al. 2021), our predictions do not support a direct mitochondrial localization. We also confirmed localization predictions about clade B by microscopy analysis of *L. interrogans* HDAC11 that belongs to clade B and was observed in the cytoplasm and nucleus.

The complex phylogenetic distribution of Class IV HDACs likely reflects a combination of evolutionary processes, including eukaryogenesis, endosymbiogenesis, endosymbiotic gene transfer, paralog loss, and vertical inheritance (Ledent and Vervoort 2006; Cavalier-Smith 2013; Kelly 2021; Lopez-Garcia and Moreira 2023). Based on our results, we propose that Class IV HDACs in all organisms are of prokaryotic origin and that clades A and B arose by gene duplication in a prokaryotic ancestor. The absence of one or both homologs in some bacterial and archaeal lineages likely results from lineage-specific gene losses. Our hypothetical evolutionary scenario (Figure S42) suggests that acetoin utilization proteins were present before the subdivision into HDAC classes, representing an ancestral group to Class IV HDACs.

Both clades A and B contain bacterial (ABAC and BBAC) and archaeal (AARC and BARC) subclades (Figure S42). Members of these subclades later diverged to give rise to present-day homologs in bacterial, archaeal, and eukaryotic species, showing signatures of early eukaryogenesis and endosymbiosis. In clade A, primary endosymbiosis with cyanobacteria or alphaproteobacteria, followed by endosymbiotic gene transfer and consequent subcellular targeting, produced plastid-, mitochondrial-, or dual-targeted enzymes in streptophytes, chlorophytes, rhodophytes, cryptophytes, haptophytes, SAR taxa (including alveolates), and some metazoans (ABAC green and brown groups; Figure S42; E/A1 in Figure S43). Supporting this interpretation, comparative analyses of Zn^2+^-dependent histone deacetylases in plants show that plant HDA2, a Class IV HDAC, clusters with cyanobacterial HDACs, consistent with an endosymbiotic contribution to nuclear Class IV repertoires (Yruela et al. 2021). Additional clade A HDACs (E/A2 in Figure S43) likely originated during eukaryogenesis (Byun-McKay and Geeta 2007) or were laterally transferred from bacterial donors, with subsequent retargeting to plastids, mitochondria, or both organelles in streptophytes, rhodophytes, chlorophytes, SAR taxa, and *Naegleria* (Discoba) (ABAC blue group; Figure S42; E/A2 left in Figure S43). Similarly, organellar targeting of archaeal-derived HDACs produced mitochondrial-targeted enzymes in rhodophytes. Complex (secondary and higher-order) endosymbioses spread the rhodophyte enzyme to organisms with rhodophyte-derived complex plastids such as SAR members, cryptophytes, and haptophytes (AARC groups; Figure S42; E/A2 right in Figure S43), where dual localization of enzymes to mitochondrion and plastid evolved. Parallel evolutionary processes in clade B’s archaeal and bacterial subclades (BARC and BBAC; Figure S42; E/B1 in Figure S43) have contributed to the present-day diversity. Conservation of paralogs from both clades, combined with gradual gene loss, has left some species with multiple Class IV HDAC genes and others with only one paralog (Table S1).

The mitochondrial or plastid localization of clade A enzymes likely reflects the acquisition of targeting presequences suited to organellar metabolic needs. Gain or loss of transit peptides or bipartite targeting sequences is common, and such signals can evolve rapidly. Signal peptides evolve on average twice as fast as mature proteins and up to five to six times faster than random sequences (Christian et al. 2020). Transit peptides may arise via duplication, horizontal gene transfer, exon shuffling, or other mechanisms and can also evolve *de novo* through random insertions or deletions (Pujol et al. 2007; Tonkin et al. 2008). Chloroplast transit peptides, in particular, exhibit low sequence conservation and often arise *de novo* relative to conserved catalytic domains (Tomcala et al. 2020). Changes in targeting sequences can alter subcellular localization, potentially conferring new functions without modifying the mature protein (Tonkin et al. 2008). Such shifts may be especially frequent in photosynthetic eukaryotes, which contain chloroplasts and other specialized organelles (Kastaniotis et al. 2017).

We hypothesize that the diversification of Class IV HDACs may be linked to differences in FAS pathways. In eukaryotes, *de novo* FAS occurs in the cytoplasm (FAS type I; FASI) and mitochondria (FAS type II; FASII) of heterotrophs, whereas in phototrophs, FASII is mainly plastid-localized (Lin et al. 2016; Cecchin et al. 2019). Heterotrophs and phototrophs differ in the origin and genetic complexity of the enzymes involved, pathway localization, and the chain length and type of fatty acids produced. Notably, HDAC localization is consistent with FAS type: Clade A HDACs are targeted to mitochondria/plastids alongside FASII in phototrophs, while clade B HDACs localize to the cytoplasm alongside FASI in heterotrophs. Some phototrophs, such as *Chlorella* (chlorophytes) and *Vitrella* (Alveolata: Apicomplexa), possess both pathways, which are differentially regulated under varying light conditions (Tomcala et al. 2020) (Jiang et al. 2014; Cecchin et al. 2019), and encode multiple HDACs with predicted dual localization (Tables S1 and S2). In contrast, apicomplexans lacking an apicoplast, such as *Cryptosporidium* spp., also lack *de novo* FAS (Cecchin et al. 2019), and no clear Class IV HDAC homologs were detected in our dataset.

Functional parallels exist with Class I HDAC3, which deacetylates fatty acid synthase (FASN), the terminal enzyme in lipogenesis (Kutil et al. 2018), and regulates peroxisome proliferator-activated receptor gamma (PPARγ) in adipocytes, thereby influencing glucose and fatty acid metabolism (Katoh and Standley 2013). HDAC11, the only Class IV HDAC in vertebrates, acts as a long-chain fatty acid deacylase (Kutil et al. 2018; Moreno-Yruela et al. 2018; Cao et al. 2019) and is strongly inhibited by physiological concentrations of free fatty acids and metabolic intermediates, suggesting a role as a fatty acid metabolism sensor (Eme et al. 2017). The enzyme has been implicated in processes related to metabolic homeostasis and obesity (Nunez-Alvarez and Suelves 2022). Human HDAC11 may also deacylate proteins nonenzymatically modified by reactive fatty acyl-CoAs, bind or transport fatty acids, or hydrolyze acyl groups from small molecules such as metabolites and metabolic intermediates (Blair et al. 2005; Hai et al. 2017; Burger and Chory 2018). Such functions could extend to other Class IV HDAC homologs, with differences in fatty acid modulation reflecting the evolutionary divergence of clades A and B. However, direct evidence for co-expression or conserved genetic linkage between Class IV HDAC and FAS genes is currently lacking, and substrate specificity and mechanistic links to the FAS pathway remain to be experimentally established. Accordingly, this association should be regarded as hypothesis-generating rather than causal.

**Figure 6:**
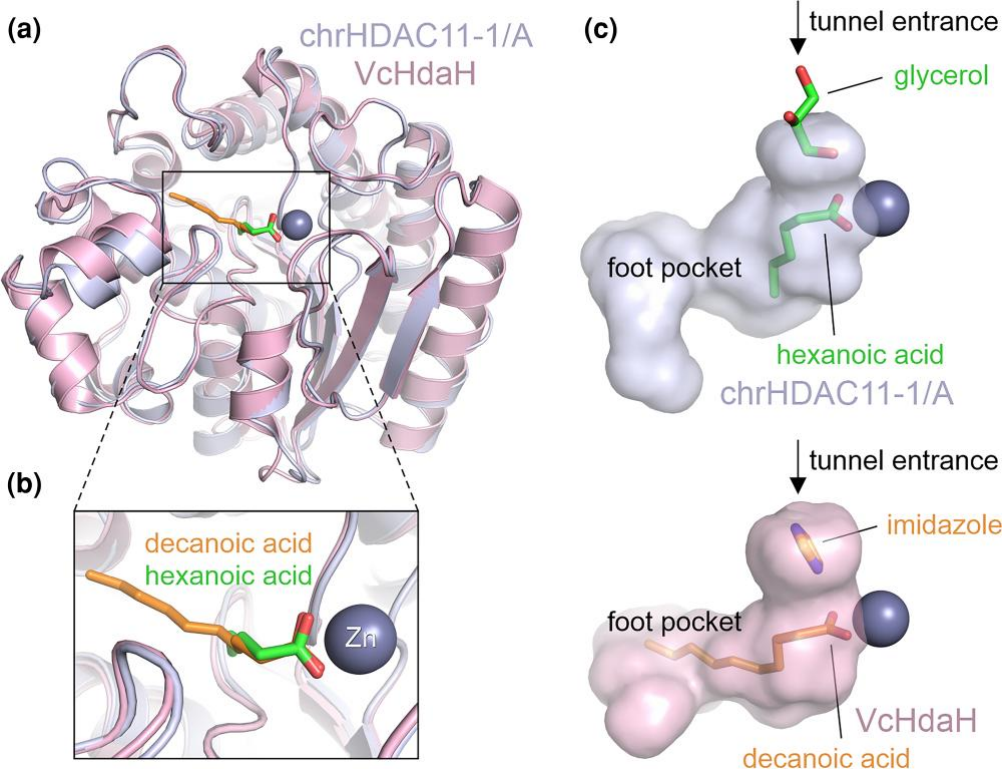
Structural comparison of chrHDAC11-1/A and HDAC11 from *V. Cholerae* (VcHdaH). (a) Structural superposition of the VcHdaH structure (pink; PDB ID: 9GKY; Graf et al. 2024) and our chrHDAC11-1/A structure (gray) shows their close structural similarity, with an RMSD of 0.68 Å over 252 corresponding Cα atoms. (b) Zoomed-in view of the fatty acids bound in the active site of the superposed structures with the carboxylate groups interacting with the zinc ion in the catalytic site. Decanoic acid (yellow) was observed in the VcHdaH foot pocket, whereas hexanoic acid (green) was found in the foot pocket of chrHDAC11-1/A. (c) View of the interior surface of the proteins. Glycerol (green) and imidazole (yellow) likely originating from crystallization buffers are shown in the entrance tunnel while hexanoic (green) and decanoic (yellow) acid, which likely represent captured endogenous or product-like species, extend toward foot pocket.

Recent work by Graf et al. (2024) described the structure and biochemical characterization of the bacterial Class IV HDAC VcHdaH from *Vibrio cholerae*. In our phylogenetic analysis, VcHdaH groups with clade A HDAC11s. In agreement with our data for clade A enzymes, Graf et al. did not observe robust deacylase activity in their substrate screens and detected only very weak de-decanoylase activity at a relatively high enzyme concentration (1 µM), supporting the view that clade A HDAC11s show little or no activity toward the peptidic acyl substrates tested so far under standard assay conditions. Structurally, the apo VcHdaH structure superposes well with our chrHDAC11-1/A structure, with an RMSD of 0.68 Å over 252 corresponding Cα atoms (Fig. 6a). Notably, Graf et al. observed decanoic acid bound in the VcHdaH foot pocket, whereas we identified hexanoic acid in the foot pocket of chrHDAC11-1/A (Fig. 6b). Because neither ligand was added during purification or crystallization, both most likely represent captured endogenous or product-like species. Together, these observations suggest that clade A HDAC11s, although inactive toward the tested acylated peptides, may nevertheless accommodate certain short- to medium-chain fatty acids within the foot pocket, likely aided by conformational plasticity of pocket-lining side chains. Interestingly, imidazole was modeled in the entrance tunnel of VcHdaH, whereas in our chrHDAC11-1/A structure, we observed glycerol at a comparable position (Fig. 6c), both likely reflecting molecules introduced during crystallization. Consistent with this interpretation, although hexanoic acid was identified in the foot pocket of chrHDAC11-1/A, we were unable to detect hydrolysis of peptide-based hexanoyl-lysine substrates or of a small set of additional hexanoylated candidate substrates. Together, these observations support the idea that bulkier ligands can access the active-site tunnel and further suggest that clade A HDAC11s may act on an alternative, as yet unidentified, nonpeptidic substrate class. Defining these physiological substrates, however, will require dedicated future studies.

Our structural and biochemical analyses show that, despite a conserved catalytic core, clades A and B of Class IV HDACs have diverged markedly in the architecture of their active-site surroundings. Differences in electrostatic potential at the substrate entrance, loop and rim composition, and foot pocket geometry likely act as sequential “selectivity filters,” shaping substrate compatibility. In clade B, these features enable efficient hydrolysis of long-chain fatty-acylated lysines, whereas in clade A, they are consistent with the absence of peptide deacylase activity and suggest specialization toward alternative, nonpeptidic substrates. Combined with their distinct subcellular localizations and phylogenetic distributions, these structural adaptations likely reflect long-term partitioning into different biochemical niches. Together, our findings establish a revised evolutionary framework for Class IV HDACs, highlight their structural and functional diversity, and provide a basis for future studies to identify physiological substrates, clarify organelle-specific roles, and explore their impact on cellular metabolism across the tree of life.

## Materials and methods

### Phylogenetic analysis

Class IV HDAC sequences were extracted from the NCBI database. Sequences were downloaded as GenBank-annotated entries or mined from publicly available genome or transcriptome assemblies (Table 1) by a sequence homology search using the tBLASTn algorithm and an *E*-value of 10^−5^. Class IV HDACs listed in the comparative genomic study by Ledent and Vervoort (2006) were used as queries for the BLAST search. The identity of our hits was confirmed by the reciprocal BLAST searches. During data mining, we endeavored to obtain the most complete sequences, particularly at the N-termini, to facilitate reliable predictions of targeting presequences for the studied enzymes.

Two multiple alignments were performed using MAFFT version 7.450 (Katoh and Standley 2013), within Geneious Prime version 2019.0.4 (Kearse et al. 2012). Automatic selection of the alignment reconstruction algorithm was used with default settings for the gap opening penalty (1.53) and offset value (0.123). The larger alignment (Alignment 1) contained 945 sequences of all HDAC classes, ie Class I, II, and IV, with the exception of unrelated Class III NAD^+^-dependent HDACs (sirtuins). The sequences were obtained from the datasets of Ledent and Vervoort (2006) and Milazzo et al. (2020) and by additional data mining (in the case of Class IV). This alignment was used to verify the monophyly of the Class IV HDACs and to confirm the bona fide status of our sequences of Class IV HDACs. The second, targeted alignment (Alignment 2) consisted of 480 amino acid sequences of Class IV HDACs from eukaryotes, bacteria, and archaea. This final dataset included 83 “reference sequences” from Ledent and Vervoort (2006), with an additional 397 sequences to enrich the taxon sampling of previously underrepresented groups. This alignment was used to determine the phylogenetic relationships among Class IV HDACs. Nonhomologous regions, mainly at the N- and C-termini, were manually removed in Geneious Prime version 2019.0.4, resulting in a final alignment of 293 (Alignment 1) and 546 positions (Alignment 2), corresponding to the conserved histone deacetylase domain (PF00850.22). Acetoin utilization proteins (AcuC) from bacteria and archaea (17 sequences) were used as outgroup for the phylogenetic analysis with the Alignment 2, as these proteins likely preceded the subdivision into HDAC Classes and are suitable for comparative purposes (Milazzo et al. 2020). Phylogenetic trees were reconstructed using the maximum likelihood (ML) method in IQ-TREE version 1.6.12 (Trifinopoulos et al. 2016) employing the LG + I + G4 protein model selected by ModelFinder (Kalyaanamoorthy et al. 2017). Bootstrap supports were based on 1,000 replicates. Visualization and graphical representation of the resulting tree were performed in Geneious Prime version 2019.0.4 and Adobe Illustrator CS5. The original graphically adjusted phylogenetic trees are available as pdf-formatted files in Figures S2 and S3.

### Predictions of sequence features and protein targeting

The sequence logos were generated with Geneious Prime version 2019.0.4. The targeting signals and subcellular localization of Class IV HDACs were predicted from the complete protein sequences using TargetP version 2.0 (Armenteros et al. 2019), SignalP versions 3.0 and 6.0 (Bendtsen et al. 2004; Teufel et al. 2022), iPSORT (Bannai et al. 2002), DeepLoc version 2.0 (Odum et al. 2024), PredSL (Petsalaki et al. 2006), Predotar version 1.04 (Small et al. 2004), and LOCALIZER version 1.0.4 (Sperschneider et al. 2017) software. In addition, ASAFind version 2.0 was used for predictions of intracellular protein targeting in diatoms and other algae with complex plastids (Gruber et al. 2015, 2025). Subcellular localizations in green algae were predicted using PredAlgo version 1.0 (Tardif et al. 2012), and ApicoAp was used to predict the apicoplast targeting of Class IV HDACs in apicomplexans (Cilingir et al. 2012). An ambiguous targeting signal is indicated for cases where an unequivocal decision about the localization could not be made due to conflicting results from the prediction software. The detailed results of the targeting signals identified for the sequences in the phylogenetic tree of Class IV HDAC can be found in Table S1.

### Cell lines and reagents

If not stated otherwise, reagents were purchased from Sigma-Aldrich (St. Louis, MO, USA). HEK293T cells adapted to grow as suspension cultures in Erlenmeyer flasks (generous gift of Dr. Ondrej Vanek, Faculty of Science, Charles University, Prague, Czech Republic) were cultivated in serum-free EX-CELL medium mixed 1:1 (v/v) with FreeStyle 293 F17 expression medium (Gibco, Thermo Fisher Scientific, Carlsbad, CA, USA). U-2 OS cell line was kindly provided by Prof. Pavel Hozak (Institute of Molecular Genetics, Prague, Czech Republic). Cells were cultivated in HEPES-supplemented high-glucose Dulbecco’s modified eagle’s medium (DMEM) supplemented with 10% fetal bovine serum (FBS) and 2 mM L-glutamine. Cells were cultured under a humidified 5% CO_2_ atmosphere at 37 °C.

### Expression plasmids and site-directed mutagenesis

Codon-optimized genes encoding individual HDAC11 homologs have been synthesized commercially (see Table 1 for sequence IDs and UniProt/NCBI codes) and cloned into the pDONR221 donor vector via the BP recombination reaction following the manufacturer’s protocol (BP Gateway cloning, Invitrogen, Thermo Fisher Scientific). Individual sequences were then transferred into the pMM322 expression plasmid (Skultetyova et al. 2017) comprising the N-terminal Twin-Strep-FLAG-HALO tag using the LR recombination reaction (Figure S5). Genes encoding *L. interrogans* (NCBI accession no. WP_000410917.1) and truncated *C. reinhardtii* (NCBI accession no. XP_042917102.1; amino acids 71-385) HDAC11 used for crystallographic studies were additionally inserted into the pEC566 plasmid as a fusion with an N-terminal His6-MBP tag (Figure S5). Site-specific mutations were introduced into HDAC11 encoding genes via a quick-change site-directed mutagenesis protocol or megaprimer protocol (Forloni et al. 2019) using gene-specific primers (Table S6). The final constructs were validated by Sanger sequencing.

Expression plasmids intended for microscopy experiments were constructed from the backbone of cumate-inducible PiggyBac transposon vector kindly provided by Dr. Kvido Strisovsky (Institute of Biochemistry and Organic Chemistry, Prague, Czech Republic) (Gourkanti et al. 2026), carrying puromycin resistance. The IRES-GFP sequence was deleted from the PiggyBac vector while restriction sites for NheI and SbfI were inserted behind the cumate switch and upstream of the EF1a promoter, respectively. Next, sequences of mScarlet3 (commercially synthesized DNA) and selected HDAC11 homologs (liHDAC11/B and chrHDAC11-1/A; sequence amplified from donor vectors) were flanked by restriction sites for KpnI, SbfI, and NheI (Table S7) and then sequentially ligated by T4 ligase into the vector backbone to build the final expression vectors liHDAC11/B-mScarlet3 and chrHD11-1/A-mScarlet3 with mScarlet3 fused to the C-terminus of the HDAC11 homolog sequence (Figure S5). The sequences of the final expression vectors were verified by Sanger sequencing.

### Live-cell imaging of HDAC11 homologs

U-2 OS cells were seeded at a density of 85,000 cells/mL in a six-well plate. After 24 h, a transfection mixture consisting of 2 µg DNA (HDAC11-mScarlet3 vectors mixed at a 3:1 ratio with the super PiggyBac transposase plasmid—a kind gift of Dr. Kvido Strisovsky), 200 µL jetOPTIMUS buffer, and 2 µL jetOPTIMUS transfection reagent (Polyplus-transfection, Illkirch, France) was prepared and incubated for 10 min at RT and then applied to the cells. Transfected cells were then continuously selected by puromycin (1 µg/mL) starting 48 h after transfection. Prior to microscopy, cells were treated with 200 µM cumate (4-isopropylbenzoic acid) for 5 d. One day before imaging, cells were seeded at a density of 8,000 cells/well in 100 µL of cumate-supplemented medium into a µ-Slide 18 Well imaging chamber with a 1.5H glass coverslip bottom (ibidi GmbH, Grafelfing, Germany). Prior to imaging, cells were treated with 10 nM MitoTracker Deep Red FM (Thermo Fisher Scientific) for 15 min, and then cultivation media was exchanged for FluoroBrite DMEM medium (Thermo Fisher Scientific) containing 10 µg/mL Hoechst 33258. Fluorescence signal of alive cells was acquired at 37 °C under 5% CO_2_ atmosphere by confocal microscope Leica TCS SP8 (Leica, Wetzlar, Germany) equipped with water immersion objective HC PL APO CS2 with 63× magnification and humidity chamber (Okolab, Pozzuoli, Italy). Images were analyzed in Fiji software (Schindelin et al. 2012) and processed in Adobe Photoshop CS4 software (Adobe Systems, San Jose, CA).

### Heterologous HDAC11 expression and purification

Constructs of HDAC11 homologs were expressed in suspension culture of HEK293T cells transiently transfected using linear polyethylene imine as described earlier (Skultetyova et al. 2017). Overexpressed proteins were purified according to a procedure published previously (Kutil et al. 2018) with minor modifications. In brief, transfected cells were suspended in lysis buffer (100 mM Tris, 10 mM NaCl, 5 mM KCl, 4 mM MgCl_2_, 10% glycerol, pH 8) supplemented with benzonase (2 U/mL; Merck Millipore, Darmstadt, Germany) and a cocktail of protease inhibitors (Roche, Basel, Switzerland). Cell suspension in a final volume of 40 mL was treated by three sonication pulses per 20 s each at 4 °C with amplitude set to 6 (probe 4420 connected to Q700 sonicator, Qsonica, Newtown, CT, USA) followed by addition of 0.2% Igepal-630. Cell lysate incubated for 20 mins at 4 °C was further supplemented by 140 mM NaCl and incubated another 20 min at 4 °C. Cell lysate was cleared by two-step centrifugation at 14,000 × *g* for 15 min at 4 °C followed by centrifugation of supernatant at 40,000 × *g* for 30 min at 4 °C. Proteins were purified using a Strep-Tactin column (IBA, Gottingen, Germany). In brief, supernatant was incubated with resin for 1 h at 4 °C in batch followed by three steps of stringent washing with 50 mM Tris, 150 mM NaCl, 10 mM KCl, and 10% glycerol, pH 8, where the second step was enriched with 3 mM ATP and 10 mM MgSO_4_. Construct was eluted by wash buffer supplemented with 3 mM desthiobiotin (IBA), concentrated to 1 to 2 mg/mL, and flash frozen in liquid nitrogen.

In lipidomic analysis and crystallographic study of *L. interrogans* and *C. reinhardtii* HDAC11, genes inserted in pEC566 vector were expressed in *E. coli* RIPL BL21 strain. *C. reinhardtii* HDAC11 was expressed as truncated variant containing amino acid 71-385. Briefly, cells were grown at 37 °C in LB medium supplemented with 100 µg/mL ampicillin up to OD_600_ 0.6. Cell culture was then equilibrated to 18 °C and supplied with 1 µM zinc acetate. Protein expression of liHDAC11 and chrHDAC11 was induced by 0.5 and 0.05 mM IPTG, respectively, and run 20 h at 18 °C. Cells were then harvested by centrifugation at 6,000 × *g* for 10 min at 4 °C, resuspended in 40 mM Na_2_HPO_4_, 10 mM NaCl, 5 mM KCl, 4 mM MgCl_2_, and 10% glycerol, pH 8, and supplemented with benzonase (2 U/mL; Merck, Darmstadt, Germany) and a cocktail of EDTA-free protease inhibitor (Roche Diagnostics GmbH, Mannheim, Germany). Cells were lysed by passing three rounds of homogenization in high-pressure homogenizer EmulsiFlex-C3 (Avestin, Inc., Ottawa, Canada) set to 1,100 bar. Lysate was further supplemented by 0.1% CHAPS detergent, incubated for 10 min at 4 °C, then supplemented by 140 mM NaCl, and incubated for another 10 min at 4 °C. Cell lysate was cleared by two-step centrifugation at 14,000 × *g* for 20 min at 4 °C followed by centrifugation of supernatant at 40,000 × *g* for 40 min at 4 °C. The supernatant was filtered and supplemented with 10 mM imidazole, and recombinant protein was purified using Ni-NTA column (Ni-NTA Superflow, IBA) with the help of batch incubation run for 1 h at 4 °C. The column was stringently washed by 20 mM Na_2_HPO_4_, 150 mM NaCl, 100 mM KCl, 10 mM imidazole, and 5% glycerol, pH 8, in three subsequent steps where the second step was enriched with 3 mM ATP and 10 mM MgSO_4_. Construct was eluted by 80 mM imidazole dissolved in wash buffer. Purified protein was concentrated to 2 mg/mL, mixed with in-house produced TEV protease in a molar ratio of 20:1 and simultaneously dialyzed overnight against an excess of 20 mM Na_2_HPO_4_, 150 mM NaCl, 100 mM KCl, and 5% glycerol, pH 8, using SnakeSkin dialysis tubing with molecular weight cut off 7 kDa (Thermo Fisher Scientific). Recombinant HDAC11 and His6-MBP tag were further purified by affinity chromatography using Ni-NTA column that was followed by size exclusion chromatography using a HiLoad 16/600 Superdex 75 pg preparative size exclusion chromatography column (Cytiva, Marlborough, MA, USA) where the buffer consisting of 25 mM Tris-HCl, 140 mM NaCl, 100 mM KCl, and 3% glycerol, pH 8, was used as a mobile phase. Purified HDAC11 variants were concentrated to 10 mg/mL, frozen in liquid nitrogen and stored at −80 °C.

### Quantification of HDAC11 deacylase activity

The enzymatic activity of HDAC11 homologs was assessed using a panel of 17 Abz-labeled peptide substrates carrying diverse acyl modifications (Abz-SRGGK(acyl)FFRR-NH_2_; Figure S7). Assays were performed as described previously (Kutil et al. 2018), with minor modifications. Purified proteins (concentrations ranging from 2 to 2,000 nM) were incubated with 10 µM substrate in 50 µL of assay buffer [1× phosphate-buffered saline (PBS) supplemented with 2 mg/mL BSA, pH 7.4] in 96-well plates. Reactions were carried out at 37 °C for 30 min with shaking at 500 rpm and were quenched by adding 5 µL of 5% acetic acid. Precipitated proteins (HDAC11 and BSA) were removed by centrifugation at 2,000 × *g* for 15 min at room temperature. Reaction mixtures were analyzed by reverse-phase high-performance liquid chromatography (RP-HPLC) using a Shimadzu Prominence system equipped with an RF-20A XS fluorescence detector (λex = 320 nm, λem = 420 nm) and a Kinetex 2.6 μm Polar C18 column (50 × 3.0 mm, Phenomenex, Torrance, CA, USA). Solvent A was water with 0.1% trifluoroacetic acid (TFA), and Solvent B was acetonitrile with 0.1% TFA.

Two gradient methods were used based on the substrate. (i) For long fatty-acylated peptides (dodecanoyl, myristoyl, palmitoyl, stearoyl), the following program was used: 0 to 3.00 min linear gradient from 15% to 25% B; 3.00 to 3.05 min ramp to 100% B; 3.05 to 7.65 min 100% B; 7.65 to 7.70 min return to 15% B; 7.70 to 12.00 min re-equilibration at 15% B. (ii) Remaining substrates: 0 to 7.00 min linear gradient from 15% to 45% B; 7.00 to 7.05 min ramp to 100% B; 7.05 to 7.65 min 100% B; 7.65 to 7.70 min return to 15% B; 7.70 to 12.00 min re-equilibration at 15% B. Flow rate was maintained at 0.6 mL/min. Substrate conversion was quantified by calculating the ratio of product peak area to the sum of product and substrate peak areas from fluorescence chromatograms. These values were normalized to protein concentration and visualized as a heatmap using GraphPad Prism 7.

### Crystallization and data collection

Crystallization conditions for liHDAC11/B and chrHDAC11-1/A truncated variant were identified using commercial sparse-matrix screens (BCS Screen and PACT Premier, Molecular Dimensions Ltd., Rotherham, UK) by the sitting-drop vapor diffusion method run at 20 °C. Three-dimensional crystals of liHDAC11/B reaching 200 µm were formed in sitting drop of the screen seeded by broken plate crystal of the same protein. The drop consisted of reservoir condition (0.1 M CaCl_2_, 0.1 M MgCl_2_, 0.1 M PIPES pH 7.0, 5.625% PEG 3350, 5.625% PEG 4000, 5.625% PEG 2000, 5.625% PEG 5000 MME) and solution containing seeding crystal [0.1 M HEPES pH 7.4, 0.1 M MgCl_2_, 0.1 M NaCl, 25% (w/v) PEG 3350] mixed at the 5:1:5 ratio with purified protein concentrated to 9 mg/mL. Prior vitrification crystals were cryoprotected by reservoir solution supplemented with 25% glycerol. Plate crystals of chrHDAC11-1/A grew to 200 µm in hanging drops formed by mixing the enzyme (8 mg/mL) with reservoir solution (0.1 M Bis-Tris, 0.2 M NaNO_3_, 15% PEG 3350, 15% glycerol, pH 6.6) at the 1:1 ratio, and seeded by broken needle crystals of the same protein diluted in reservoir solution. Crystals were harvested, cryoprotected with reservoir solution supplemented with 20% glycerol, and vitrified in liquid nitrogen.

X-ray diffraction data were collected at 100 K at the BL14.1 beamline at BESSY II (Helmholtz-Zentrum Berlin für Materialien und Energie, Germany; Mueller et al. 2025). Data from the chrHDAC11-1/A crystal were processed using the XDSApp software package (Sparta et al. 2016). The liHDAC11/B dataset was indexed and integrated using XDS (Kabsch 2010) and scaled with Aimless (Evans 2011). Detailed crystallographic statistics are provided in Table S5.

### Structure solving and refinement

Structure of liHDAC11/B was solved using Auto-Rickshaw, an automated crystal structure determination platform (Panjikar et al. 2005). Structure of chrHDAC11-1/A was determined by molecular replacement using the model of chrHDAC11-1/A generated by AlphaFold2 server (Jumper et al. 2021) as the initial model for Phaser (McCoy et al. 2007). Iterative model building and refinement were performed using Coot (Emsley et al. 2010) and phenix.refine (Afonine et al. 2012). The quality of the final models was assessed by wwPDB OneDep system (Gore et al. 2017). Final statistics, including R-factors, resolution limits, and model geometry, are summarized in Table S5. Coordinates and structure factors have been deposited in the Protein Data Bank under accession codes 9RJE and 9RJD for chrHDAC11-1/A and liHDAC11/B, respectively.

### HDAC11 homolog modeling

The *in silico* models of protein sequences listed in Table 1 were generated using ColabFold v1.5.5 (Mirdita et al. 2022) (https://colab.research.google.com/github/sokrypton/ColabFold/blob/main/AlphaFold2.ipynb#scrollTo=ADDuaolKmjGW), accessed in January 2024. ColabFold utilizes AlphaFold2 for protein structure predictions using multiple sequence alignments generated through MMseqs2. Template mode was set to pdb100, while default options were kept for all other settings. Maestro from Schrodinger 2019-1 suite (Schrodinger LLC, New York, NY, USA) was used for visualization and analysis.

### Sample preparation for GC–MS—diazomethane derivatization

To prepare the diazomethane reagent, 20 mL of 40% aqueous NaOH was mixed with 20 mL of isopropanol (Mix A). Separately, 1.5 g of Diazald (Merck, Darmstadt, Germany) was dissolved in 70 mL of diethyl ether (Mix B). While cooling Mix A on wet ice, Mix B was added dropwise under constant stirring. Diazomethane was subsequently distilled from the final reaction mixture along with diethyl ether and used immediately for derivatization. Purified protein samples (3.5 mg/mL in phosphate-buffered saline) were digested overnight at 37 °C with trypsin in weight ratio 40:1 to facilitate fatty acid release. A 2 mL aliquot of the digest was transferred to a 20 mL glass vial containing 0.5 mL of Milli-Q water supplemented with 560 mg of NaCl and 5 µL of 36% HCl. After vigorous vortexing, fatty acids were extracted in three consecutive steps: twice with 5 mL of methyl tert-butyl ether (MTBE) and once with 5 mL of a methanol:chloroform mixture (2:1, v/v). The combined organic layers were collected using a glass Pasteur pipette, pooled, and evaporated under a stream of argon to approximately 1 mL. A few drops of freshly prepared diazomethane in diethyl ether were then added gradually with gentle handshaking until the solution turned pale yellow, indicating saturation. Excess diazomethane was removed under argon, and the sample was further concentrated to ∼1 mL for GC–MS analysis.

### Analysis of long-chain fatty acids methyl esters (FAMEs) using GC–MS

FAMEs were analyzed using a Pegasus 4D GCxGC–MS system (LECO Corp., St. Joseph, MI, USA) equipped with electron ionization. Chromatographic separation was performed in one dimensional mode using a TR-FAME capillary column (58 mm × 0.25 mm, Thermo Fisher Scientific). The inlet temperature was set to 250 °C, with a split ratio of 3:1 and constant helium flow of 1.2 mL/min. The secondary oven was maintained 15 °C above the primary oven. The primary oven was programmed as follows: an initial hold at 90 °C for 1 min, ramped at 10 °C/min to 140 °C, and then at 4 °C/min to 190 °C, followed by a final ramp of 8 °C/min to 250 °C, where it was held for 12 min. A temperature offset of +5 °C was applied to the secondary column. The transfer line temperature was maintained at 280 °C, and the ion source was held at 250 °C. Mass spectra were acquired over a scan range of 29 to 600 m/z.

Data were processed using ChromaTOF software (v4.72). FAMEs were identified based on a combination of retention times and mass spectral data, using the NIST MS 2.2 library and confirmed with commercial standards (GLC-744, Avanti, Merck; cis-Dodec-5-enoic acid, ChemCruz, Santa Cruz Biotechnology, Dallas, TX, USA).

### Analysis of shorter-chain fatty acids using GCxGC–MS

Fatty acids in proteinaceous samples were derivatized using methyl chloroformate (MCF) in isopropanol. A 200 µL aliquot of the sample, consisting of 0.75 mg of protein dissolved in phosphate-buffered saline (PBS), was mixed with 150 µL of isopropanol and 100 µL of pyridine (both HPLC grade, Sigma-Aldrich). A total volume of 80 µL of MCF (99%, Sigma-Aldrich) was subsequently added to the mixture to initiate derivatization. MCF was added in four sequential portions of 20 µL each, with vortexing after every addition. All the operations described were carried out using solvents and reagents chilled on ice. After the derivatization process was completed, 2 mL of water (LC–MS grade) was added to the solution. A PDMS-coated stir bar (Twister, 1 cm in length, 0.5 mm film thickness) was then inserted into the mixture. The vial was placed on a magnetic stirrer and stirred for 60 min to facilitate efficient extraction of the derivatized compounds.

The analysis was carried out using a comprehensive two-dimensional gas chromatography system (GC × GC) consisting of an Agilent 8890 gas chromatograph (Agilent technologies, Santa Clara, CA, USA) coupled to a LECO BT time-of-flight mass spectrometer (LECO Corp.). The separation was performed using a primary Rxi-5MS column (30 m length, 0.25 mm internal diameter, 0.25 µm film thickness) and a secondary Rxi-17Sil MS column (1.5 m length, 0.25 mm internal diameter, 0.25 µm film thickness). Helium was used as the carrier gas at a constant flow rate of 1.0 mL/min. The inlet system consisted of a GERSTEL Thermal Desorption Unit (TDU; GERSTEL GmbH, Mulheim an der Ruhr, Germany) combined with a programmed temperature vaporizing (PTV) injector operating in solvent vent mode. The TDU was programmed from 40 °C (held for 0.5 min) to 290 °C at a heating rate of 60 °C/min, followed by a 5 min hold. The PTV was programmed from 10 °C (held for 0.2 min) to 290 °C at a heating rate of 10 °C/s, with a final hold of 3.8 min. During solvent venting, the flow was set to 50 mL/min at 3 psi, with an inlet purge time of 4 s and total splitless inlet flow of 11 mL/min. The oven temperature program was as follows: initial temperature of 40 °C (held for 4 min), ramped at 5 °C/min to 200 °C, and then further increased at 15 °C/min to a final temperature of 320 °C (held for 1 min). Data evaluation was performed using ChromaTOF software. Quantitative comparison was carried out using the peak areas of short-chain fatty acid propyl esters in the analyzed samples.

## Supplementary Material

msag150_Supplementary_Data

## Data Availability

The data underlying this article are available in the article and in its online supplementary material. Structural data are available in the RCSB Protein Data Bank under PDB Code ID: 9RJD and 9RJE.
