# Supplementary material for: Functional divergence and structural changes of Class IV histone deacetylases (HDACs) across the tree of life
Source: Mol Biol Evol. 2026 Jun 17;43(7):msag150. doi: 10.1093/molbev/msag150 (PMC13325672; doi:10.1093/molbev/msag150)
Supplement: msag150_Supplementary_Data [file msag150_supplementary_data.zip › SI_Novakova_HD11.pdf]

Supplementary material

Functional Divergence and Structural Changes of class IV Histone Deacetylases (HDACs) Across the Tree of Life

Zora Nováková[1†], Pavla Bartošová-Sojková[2†*], Julia Kudláčová[1], Fady Baselious[3], Zsófia Kutilová[1], Pavlína Jaklová[1], Marat Meleshin[4], Lucia Motlová[1], Andrea Schenkmayerova[1], Vladimír Vrkoslav[5], Štěpán Strnad[5], Natan Horáček[5], Ansgar Gruber[2,6], Petr Žáček[7], Sebastian Kroll[1], Barbora Havlínová[1], Markéta Ondráková[1], Růžena Tučková[1], Tereza Krunclová[1], Josef Cvačka[5], Miroslav Oborník[2,6], Mike Schutkowski[4], Wolfgang Sippl[3], Cyril Bařinka[1*]

[1] Institute of Biotechnology of the Czech Academy of Sciences, BIOCEV, Prumyslova 595, 252 50 Vestec, Czech Republic

[2] Institute of Parasitology, Biology Centre of the Czech Academy of Sciences, Branisovska 31, 370 05, Ceske Budejovice, Czech Republic

[3] Department of Medicinal Chemistry, Institute of Pharmacy, Martin-Luther-University of Halle-Wittenberg, 06120 Halle (Saale), Germany

[4] Charles Tanford Protein Center, Department of Enzymology, Institute of Biochemistry and Biotechnology, Martin-Luther-University of Halle-Wittenberg, 06120 Halle (Saale), Germany

[5] Institute of Organic Chemistry and Biochemistry, Czech Academy of Sciences, Flemingovo nam. 2, Prague 160 00, Czech Republic

[6] Faculty of Science, University of South Bohemia, Branisovska 1760, 370 05, Ceske Budejovice, Czech Republic

[7] OMICS Mass Spectrometry Core Facility, Biology Department, Faculty of Science, Charles University, BIOCEV, Prumyslova 595, Vestec 25242, Czech Republic

List of content

Table S1 (file). Predicted targeting signals and subcellular localizations of taxa used for phylogenetic tree reconstruction in Fig. 1.

Table S2. Representative examples of multiple class IV HDAC paralogs in selected species, showing distinct targeting signals and predicted subcellular localizations.

Table S3 (file). Substrate preference of HDAC11 homologs.

Table S4 (file). Conservation of amino acid residues within clades A and B.

Table S5: Data collections and refinement statistics.

Table S6: List of primers used for site-directed mutagenesis

Table S7: Primers and PCR conditions used for construction of cumate-inducible expression vectors

Figure S1. Unrooted circle-formatted maximum likelihood phylogenetic tree of all HDACs classes.

Figure S2 (file). Maximum likelihood phylogenetic tree of all HDACs classes.

Figure S3 (file). Maximum likelihood phylogenetic tree of class IV HDACs.

Figure S4. Sequence logos showing conservation of four catalytic motifs HDAC classes, as identified from sequences present the phylogenetic tree in Figure S1 and S2.

Figure S5. Schematic representations and sequences of recombinant constructs.

Figure S6. Purification of HDAC11 variants from HEK293T lysates.

Figure S7. Schematic representation of acyl-peptide library used for enzymatic activity profiling.

Figure S8. HPLC chromatogram of monitoring HDAC11 enzymatic activity.

Figure S9 (file). Comparative model of cvHDAC11/A generated *in silico* for structural analyses of conserved motifs.

Figure S10 (file). Comparative model of gvHDAC11/A generated *in silico* for structural analyses of conserved motifs.

Figure S11 (file). Comparative model of chrHDAC11-1/A generated *in silico* for structural analyses of conserved motifs.

Figure S12 (file). Comparative model of nvHDAC11/A generated *in silico* for structural analyses of conserved motifs.

Figure S13 (file). Comparative model of lmHDAC11/A generated *in silico* for structural analyses of conserved motifs.

Figure S14 (file). Comparative model of psHDAC11/A generated *in silico* for structural analyses of conserved motifs.

Figure S15 (file). Comparative model of chrHDAC11-2/A generated *in silico* for structural analyses of conserved motifs.

Figure S16 (file). Comparative model of eaHDAC11/A generated *in silico* for structural analyses of conserved motifs.

Figure S17 (file). Comparative model of acibHDAC11/B generated *in silico* for structural analyses of conserved motifs.

Figure S18 (file). Comparative model of liHDAC11/B generated *in silico* for structural analyses of conserved motifs.

Figure S19 (file). Comparative model of cwaHDAC11/B generated *in silico* for structural analyses of conserved motifs.

Figure S20 (file). Comparative model of dbHDAC11/B generated *in silico* for structural analyses of conserved motifs.

Figure S21 (file). Comparative model of pbHDAC11/B generated *in silico* for structural analyses of conserved motifs.

Figure S22 (file). Comparative model of albaHDAC11/B generated *in silico* for structural analyses of conserved motifs.

Figure S23 (file). Comparative model of ttHDAC11/B generated *in silico* for structural analyses of conserved motifs.

Figure S24 (file). Comparative model of chrHDAC11/B generated *in silico* for structural analyses of conserved motifs.

Figure S25 (file). Comparative model of ceHDAC11/B generated *in silico* for structural analyses of conserved motifs.

Figure S26 (file). Comparative model of atHDAC11/B generated *in silico* for structural analyses of conserved motifs.

Figure S27 (file). Comparative model of lmHDAC11/B generated *in silico* for structural analyses of conserved motifs.

Figure S28 (file). Comparative model of dmHDAC11/B generated *in silico* for structural analyses of conserved motifs.

Figure S29 (file). Comparative model of agHDAC11/B generated *in silico* for structural analyses of conserved motifs.

Figure S30 (file). Comparative model of nvHDAC11/B generated *in silico* for structural analyses of conserved motifs.

Figure S31 (file). Comparative model of hsHDAC11/B generated *in silico* for structural analyses of conserved motifs.

Figure S32 (file). Comparative model of mmHDAC11/B generated *in silico* for structural analyses of conserved motifs.

Figure S33 (file). Comparative model of ssHDAC11/B generated *in silico* for structural analyses of conserved motifs.

Figure S34. The internal pocket of representative clade A and B enzymes.

Figure S35. Visualization of the foot pocket of chrHDAC11-1/A and liHDAC11/B.

Figure S36. Electrostatic potential of clade B HDACs.

Figure S37. Electrostatic potential of clade A HDACs.

Figure S38. Hydrophobicity analysis of surface and the internal pockets of representative clade A and B enzymes.

Figure S39. Circle-formatted maximum likelihood phylogenetic tree of 480 class IV HDACs.

Figure S40. Site-directed mutagenesis of the foot pocket gatekeeper residues.

Figure S41. Identification of fatty acids present in HDAC11 preparations.

Figure S42. Hypothetical evolution of class IV HDACs.

Figure S43. The proposed evolutionary scenario of class IV superimposed on a phylogenetic tree.

Supplementary Material and Methods

Table S1 (file). Predicted targeting signals and subcellular localizations of taxa used for phylogenetic tree reconstruction in Fig. 1.

Table S2. Representative examples of multiple class IV HDAC paralogs in selected species, showing distinct targeting signals and predicted subcellular localizations. mTP - mitochondrial transit peptide; cTP - chloroplast transit peptide.

**Taxon name**	**Taxonomic group**	**HDAC IV clade**	**Targeting signal/Predicted localization**
Bryopsis sp.	Chlorophyta	B	None/cytoplasm
		B	mTP, cTP/ mitochondrion, plastid
Edaphochlamys debaryana	Chlorophyta	A	mTP, cTP/mitochondrion
		B	None/cytoplasm
Chlamydomonas incerta	Chlorophyta	A	mTP, cTP/plastid
		B	None/cytoplasm
Ceratodon purpureus	Streptophyta	A	mTP/mitochondrion
		B	None/cytoplasm
		B	cTP/plastid
Physcomitrium patens	Streptophyta	A	mTP/mitochondrion
		B	None/cytoplasm
		B	None/cytoplasm
Gasterosteus aculeatus aculeatus	Metazoa	A	mTP/mitochondrion
		B	None/cytoplasm
Locusta migratoria	Metazoa	A	mTP/mitochondrion
		B	None/cytoplasm, nucleus
Vitrella brassicaformis	SAR: Alveolata	A	mTP/mitochondrion
		A	cTP/plastid

Table S3 (file). Substrate preference of HDAC11 homologs.

Table S4 (file). Conservation of amino acid residues within clades A and B.

Table S5. Data collections and refinement statistics

Data collection statistics		
HDAC11 homolog	chrHDAC11-1/A	liHDAC11/B
PDB code	9RJE	9RJD
Wavelength (Å)	0.9184	0.9184
Space group	23	1
Unit-cell parameters *a, b, c* (Å); α, β, γ (°)	80.66, 90.04, 102.97; 90.00, 90.00, 90.00	48.74, 50.37, 62.18; 88.49, 89.97, 79.72
Resolution limits (Å)	50.00-1.04 (1.10-1.04)	49.54-1.51 (1.53-1.51)
Number of unique reflections	174843 (26066)	83014 (2612)
Redundancy	12.48 (10.40)	3.7 (3.5)
Completeness (%)	97.9 (90.7)	90 (56.8)
\I/σI	13.72 (2.13)	14.1 (2.4)
R_{merge}	0.15 (1.135)	0.058 (0.518)
CC1/2	0.999 (0.741)	0.998 (0.785)
**Refinement**		
Resolution limits (Å)	32.07-1.06 (1.08-1.06)	32.19-1.51 (1.52-1.51)
Total number of reflections	166617 (10392)	83005 (1833)
Number of reflections in working set	164518(10261)	78886 (1719)
Number of reflections in test set	2099 (131)	4119 (114)
R/R_{free} (%)	14.22/16.49 (27.90/35.60)	15.61/18.41 (28.18/33.97)
Total number of non-H atoms	3079	5759
Number of non-H protein atoms	2634	5189
Number fatty acid atoms	8	28
Number of water molecules	425	522
Average B-factor ($Å^2$)	13.68	17.02
Protein atoms	9.92	15.92
Waters	30.13	27.28
Fatty acid	9.655	22.72
**[&]Ramachandran Plot (%)**		
Most favored	98	98
Additionally allowed	2	2
Disallowed	0	0 (237A/B Asp)
**R.m.s. deviations:**		
bond lengths (Å)	0.004	0.005
bond angles (°)	0.785	0.833
planarity (Å)	0.009	0.006
chiral centers ($Å^3$)	0.075	0.054
Missing residues	0	Chain A: AA1-3

* Values in parenthesis are for the highest resolution shells.

** Structures were analyzed using the wwPDB OneDep system (Gore, et al. 2017).

Table S6: List of primers used for site-directed mutagenesis

Constuct	Primer	Sequence (5' to 3')
atHDAC11/B(F199T)	forward	ATCGGCGGAGGCACTCATCACTGCACC
	reverse	GTGCAGTGATGAGTGCCTCCGCCGATG
chrHDAC11-1/A(Y357F)	forward	GTTGGTGGTGGTTTTGATGATGATCTG
	reverse	CAGATCATCATCAAAACCACCACCAAC
chrHDAC11/B(P109R)	forward	GAGAAGCTGCACCGTTTCGATGCCGG
	reverse	CCGGCATCGAAACGGTGCAGCTTCTC
hsHDAC11/B(E94G)	forward	CTACCATCACAGGAATCCCCCCCG
	reverse	CGGGGGGGATTCCTGTGATGGTAG
hsHDAC11(F141T)	forward	GTGGCACCCACCACTGCTCCAG
	reverse	TAGAAGGCACAGTCGAGG
hsHDAC11/B(P113R)	forward	GTGCTGAGGCGCCTTCGGAC
	reverse	TAGAAGGCACAGTCGAGG
liHDAC11/B(E84G)	forward	CCCAATATAGTGGACTGCCGCTGAC
	reverse	GTCAGCGGCAGTCCACTATATTGGG
liHDAC11/B(F122T)	forward	ATTGGCGGGGGCACTCATCATTCCATG
	reverse	ATGGAATGATGAGTGCCCCCGCCAATATG
liHDAC11/B(S94R)	forward	CAGATCGTGCACAGATTTGTGCTGG
	reverse	TAGAAGGCACAGTCGAGG
lmHDAC11/B(P56R)	forward	CTGCACAGGTTCGATGCCAACAAGTG
	reverse	CATCGAACCTGTGCAGCTTCTCCACG
nvHDAC11/B(F158T)	forward	ATCGGAGGAGGAACTCACCACTGCAGC
	reverse	CTGCAGTGGTGAGTTCCTCCTCCGATG
nvHDAC11/B(P53R)	forward	GGAGCGGCTGCACAGATTTGACTCCGGC
	reverse	GCCGGAGTCAAATCTGTGCAGCCGCTCC

Table S7. Primers and PCR conditions used for construction of cumate-inducible expression vectors

**Primer list**				
Gene	Primer	Sequence		
mScarlet3-C	forward primer	5'-CATGGTACCGGTGGAGGTTCTGGAGGTGGTGGATCAAGTTTAAACATGGATAGCACCGAGGCAGTGATCAAG-3'		
	reverse primer	5'-CATCCTGCAGGTTATTAGGAGCCACCGGAGCCGC-3'		
chrHDAC11-1/A	forward primer	5'-CATGCGGCCGCGCCACCATGAGCCTGGCAAGGC-3'		
	reverse primer	5'-CATGGTACCCCTCTCGCCTCTGCCCTCCC-3'		
liHDAC11/B	forward primer	5'-CATGCGGCCGCGCCACCATGGAAAAACACTTAGAGAGGATAGG-3'		
	reverse primer	5'-CATGGTACCGTCGGCAGCGAAAATTTTAATGGTGTTATAATG-3'		
**PCR reaction mixture**				
Reagent	Final concentration			
Q5 buffer	1x			
dNTPs (each)	200 µM			
forward primer	0.06 µM			
reverse primer	0.06 µM			
DNA template	1 ng/µL			
Q5 polymerase	1x			
**PCR run**				
Step	Temperature	Time	Number of cycles	
initial denaturation	98 °C	30 s	1	
denaturation	98 °C	10 s	30	
annealing	x °C	30 s		
extension	72 °C	30 s		
final extension	72 °C	10 min	1	
**Annealing temperature**				
Gene	Temperature			
mScarlet3	72 °C			
chrHDAC11-1/A	72 °C			
liHDAC11/B	69 °C			

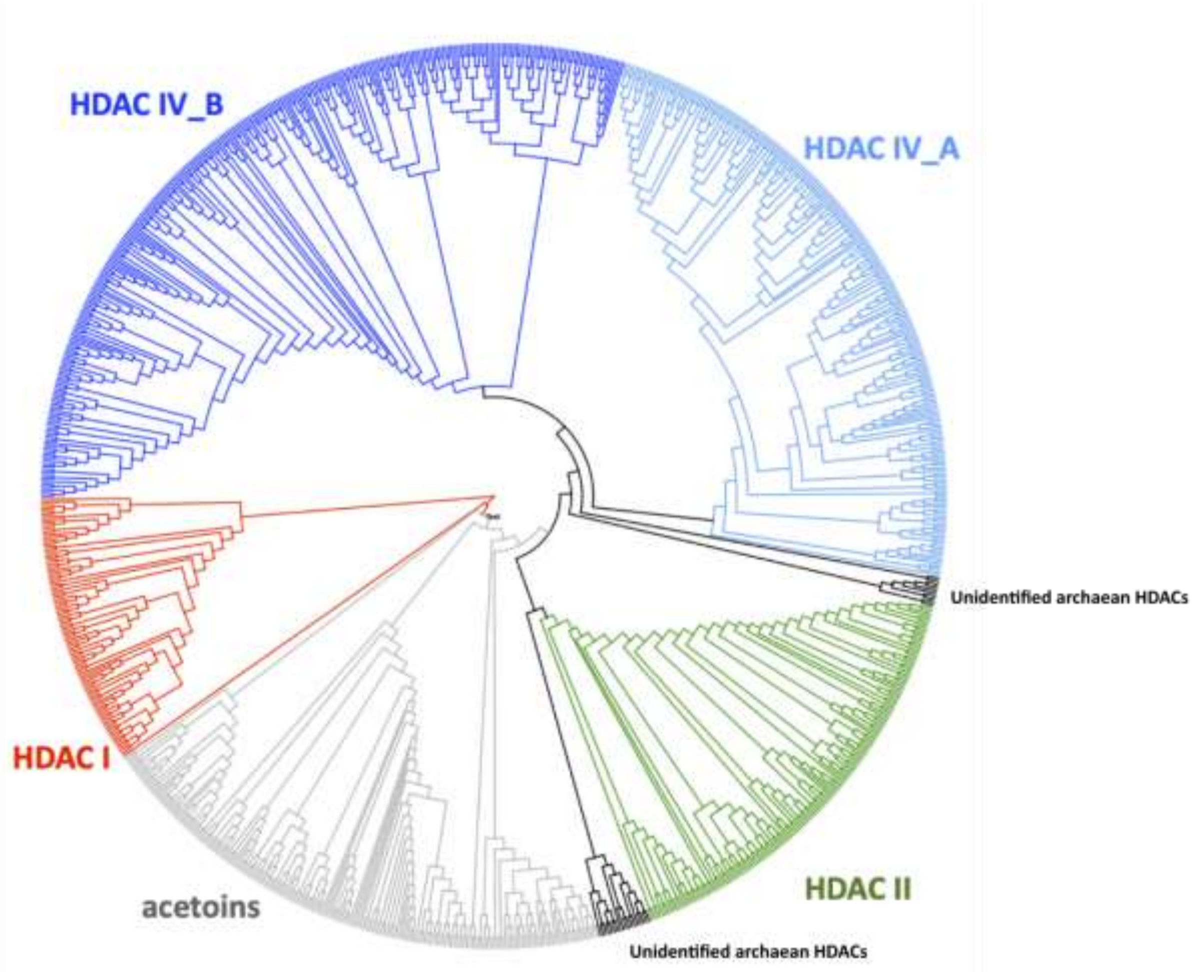

Figure S1. Unrooted circle-formatted maximum likelihood phylogenetic tree of all HDACs classes. Class IV HDACs split in two major clades, A and B.

Figure S2 (file). Maximum likelihood phylogenetic tree of all HDACs classes. Class IV HDACs split in two major clades, A and B. The bootstrap supports calculated from 1000 replicates are shown at each node.

Figure S3 (file). Maximum likelihood phylogenetic tree of class IV HDACs. Two major clades, A and B, are formed. The SH-aLRT and bootstrap supports calculated from 1000 replicates are shown at each node.

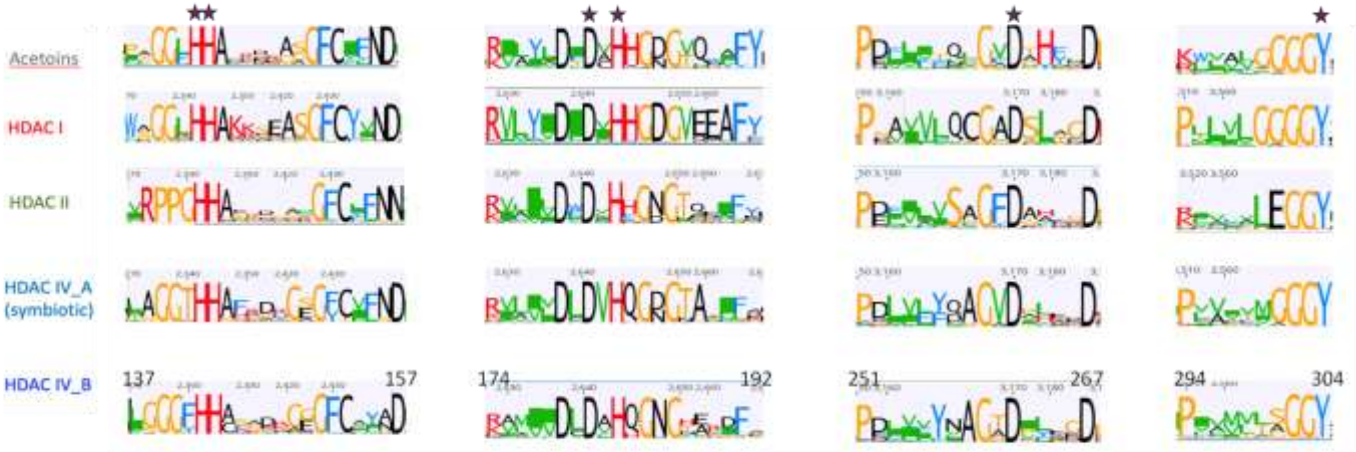

Figure S4. Sequence logos showing conservation of four catalytic motifs HDAC classes, as identified from sequences present the phylogenetic tree in Figure S1 and S2. Red asterisks mark catalytic residues. Differences are evident also between class IV A and B clades. For class IV_B, residue numbers shown above the logos correspond to human HDAC11 (NP_079103.2).

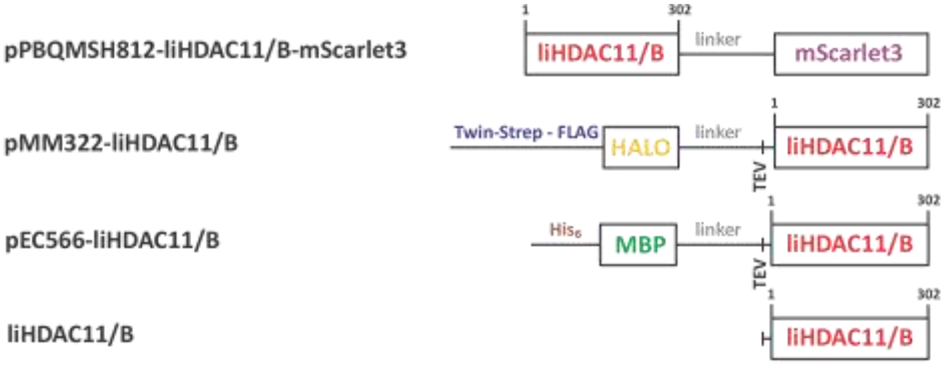

pPBQMSH812-liHDAC11/B-mScarlet3

MEKHLERIGLVYHPDYNLDLGPHVFPARKYQMVYDLVKRDSKLSNLYIYKPDLAKTKDLSLVHTQEFLDDFFSLNITERTQYSEL
PLTKQIVHSFVLAVGGTILSMELAQKYKFVYHIGGGFHHSMPDRAEGFCYLNDAAIASKLYQKEYPDKKILFIDLDLHQGNGNS
FIFQNDPDVFTFSMHQENLYPKKEKSDLDISLEEGIGDKEYLELLEKSLRKIESDFKPDLIFYIAGADPFEGDSLGDLKLTFQGLRK
RDQIVRDFAYSLNDTRVVILPAGGYAKDFYDTVTIHYNTIKIFAADGTGGGSGGGGSSLNMDSTEAVIKEFMRFKVHMEGSM
NGHEFEIEGEGEGRPYEGTQTAKLRVTKGGPLPFSWDILSPQFMYGSRAFTKHPADIPDYWKQSFPEGFKWERVMNFEDGG
AVSVAQDTSLEDGTLIYKVKLRGTNFPPDGPVMQKKTMGWEASTERLYPEDVVLKGDIKMALRLKDGGRYLADFKTTYRAK
KPVQMPGAFNIDRKLDITSHNEDYTVVEQYERSVARHSTGGSGGS

pMM322-liHDAC11/B

MASAWSHPQFEKGGGSGGGSGGSAWSHPQFEKGGSGGSDYKDDDDKGSGSGEIGTGFPFDPHYVEVLGERMHYVDVGP
RDGTPVLFLHGNPTSSYVWRNIIPHVAPTHRCIAPDLIGMGKSDKPDLGYFFDDHVRFMDAFIEALGLEEVVLVIHDWGSAL
GFHWAKRNPERVKGIAFMEFIRPIPTWDEWPEFARETFQAFRTTDVGRKLIIDQNVFIEGTLPMGVVRPLTEVEMDHYREPF
LNPVDREPLWRFPNELPIAGEPANIVALVEEYMDWLHQSPVPKLLFWGTPGVLIPPAEAARLAKSLPNCKAVDIGPGLNLLQE
DNPDLIGSEIARWLSTLEISGEPTTEDLYFQSDNAIASEFCRYPAQWRPLESSRHNQTSLYKKAGENLYFQGGGTMEKHLERIG
LVYHPDYNLDLGPHVFPARKYQMVYDLVKRDSKLSNLYIYKPDLAKTKDLSLVHTQEFLDDFFSLNITERTQYSELPLTKQIVHSF
VLAVGGTILSMELAQKYKFVYHIGGGFHHSMPDRAEGFCYLNDAAIASKLYQKEYPDKKILFIDLDLHQGNGNSFIFQNDPDV
FTFSMHQENLYPKKEKSDLDISLEEGIGDKEYLELLEKSLRKIESDFKPDLIFYIAGADPFEGDSLGDLKLTFQGLRKRDQIVRDFA
YSLNDTRVVILPAGGYAKDFYDTVTIHYNTIKIFAAD

pEC566-liHDAC11/B

MRSHHHHHHGKIEEGKLVIWINGDKGYNGLAEVGKKFEKDTGIKVTVEHPDKLEEKFPQVAATGDGPDIIFWAHDRFGGYA
QSGLLAEITPDKAFQDKLYPFTWDAVRYNGKLIAYPIAVEALSLIYNKDLLPNPPKTWEEIPALDKELKAKGKSALMFNLQEPYF
TWPLIAADGGYAFKYENGKYDIKDVGVDNAGAKAGLTFLVDLIKNKHMNADTDYSIAEAAFNKGETAMTINGPWAWSNID
TSKVNYGVTVLPTFKGQPSKPFVGVLSAGINAASPNKELAKEFLENYLLTDEGLEAVNKDKPLGAVALKSYEEELAKDPRIAAT
MENAQKGEIMPNIPQMSAFWYAVRTAVINAASGRQTVDEALKDAQTNSITSLYKKAGSENLYFQGGGMEKHLERIGLVYHP
DYNLDLGPHVFPARKYQMVYDLVKRDSKLSNLYIYKPDLAKTKDLSLVHTQEFLDDFFSLNITERTQYSELPLTKQIVHSFVLAV
GGTILSMELAQKYKFVYHIGGGFHHSMPDRAEGFCYLNDAAIASKLYQKEYPDKKILFIDLDLHQGNGNSFIFQNDPDVFTFS
MHQENLYPKKEKSDLDISLEEGIGDKEYLELLEKSLRKIESDFKPDLIFYIAGADPFEGDSLGDLKLTFQGLRKRDQIVRDFAYSLN
DTRVVILPAGGYAKDFYDTVTIHYNTIKIFAAD

Figure S5. Schematic representations and sequences of recombinant constructs. Example of liHDAC11/B homolog (red) expressed in U-2 OS cells in the fusion with mScarlet3 fluorescent protein (purple; pPBQMSH812 construct) to determine its subcellular localization. Same HDAC11 homolog was expressed in mammalian HEK293T cells (the pMM322 construct) in the form of fusion with the HALO solubility tag (yellow) and Twin-Strep and FLAG (blue) affinity tags used for purification. Recombinant proteins expressed in HEK293T cells were used to analyze their substrate specificity. The pEC566 construct comprising the MBP solubility tag (green) and the His6 affinity tag (brown) connected by the TEC-cleavable linker (grey). The recombinant liHDAC11/B protein was affinity purified and used for X-ray crystallography.

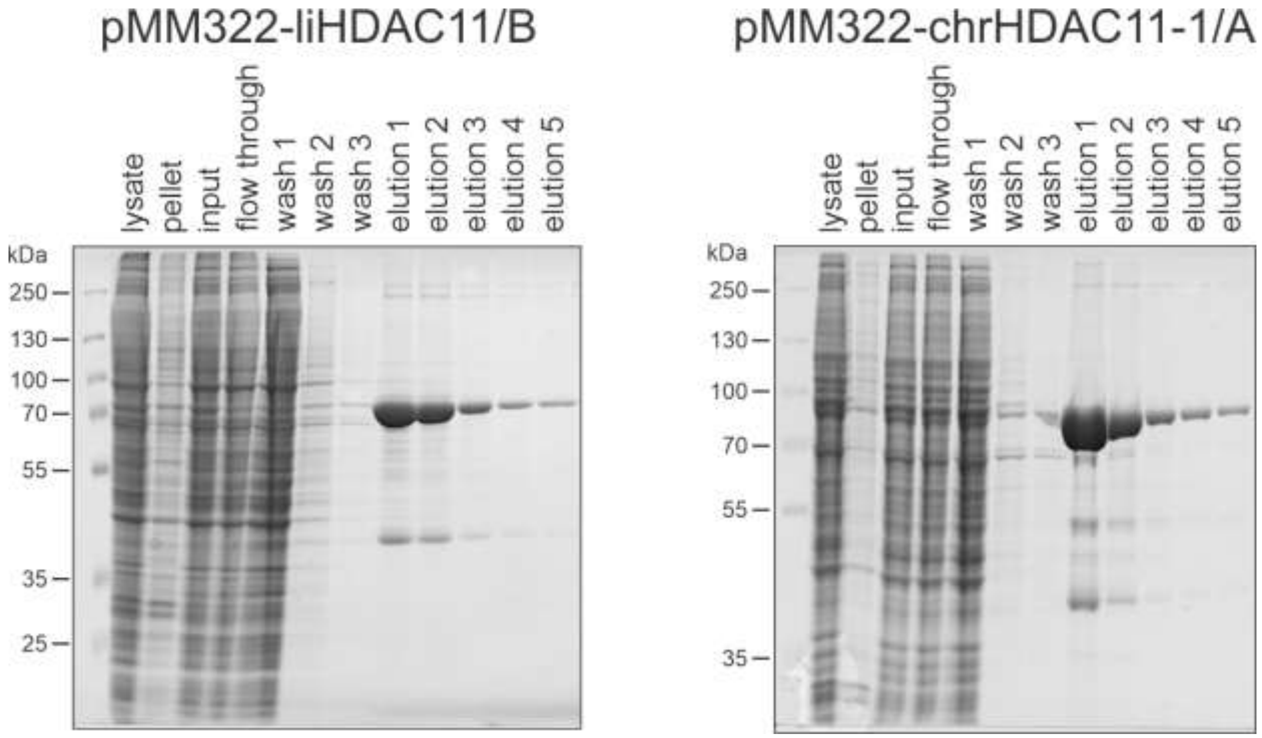

Figure S6. Purification of HDAC11 variants from HEK293T lysates. CBB-G-250-stained SDS-PAGE gels of liHDAC11/B and chrHDAC11-1/A purified via StrepTactin affinity chromatography.

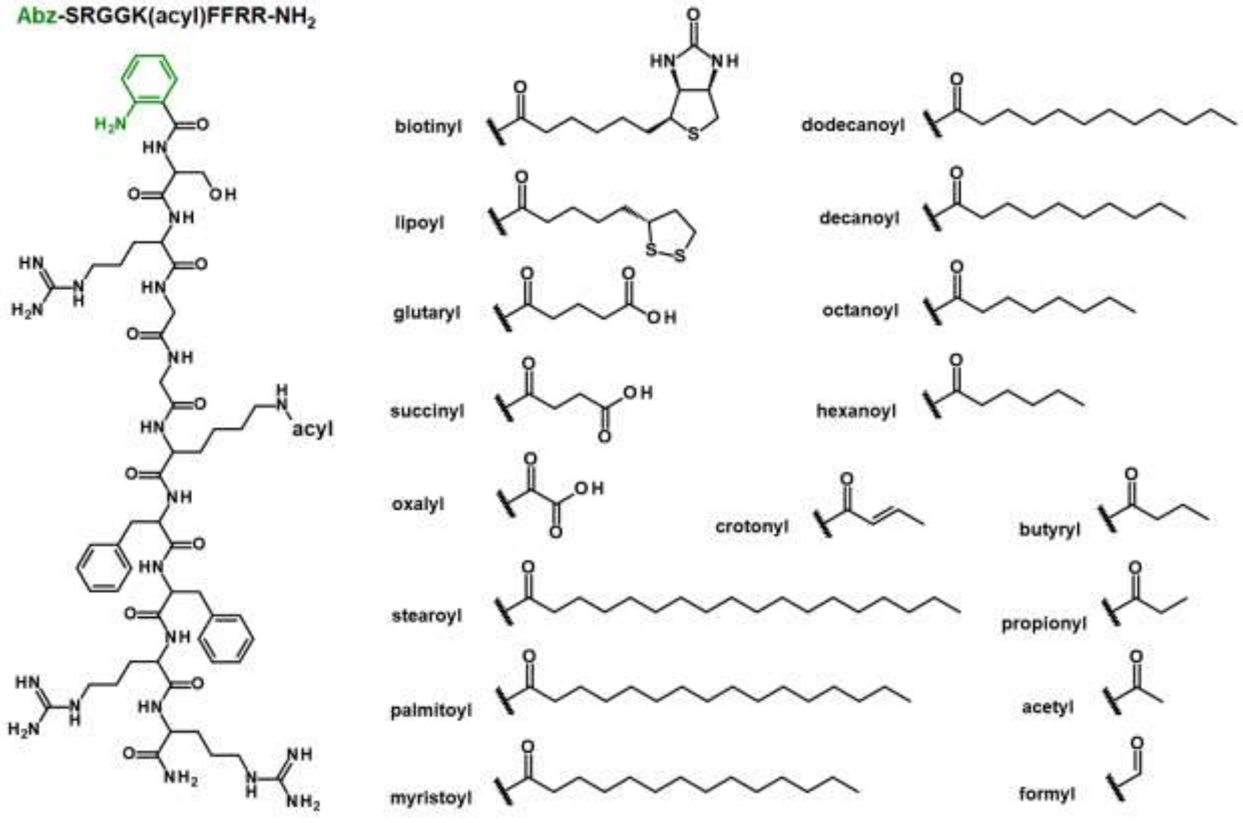

Figure S7. Schematic representation of acyl-peptide library used for enzymatic activity profiling. The peptide substrate sequence (upper left) is derivatized with acyl groups covalently attached to the ε-NH_2 group of the central lysine. The aminobezoyl functional group (green) was used for fluorescent monitoring of substrate processing by studied enzymes using HPLC.

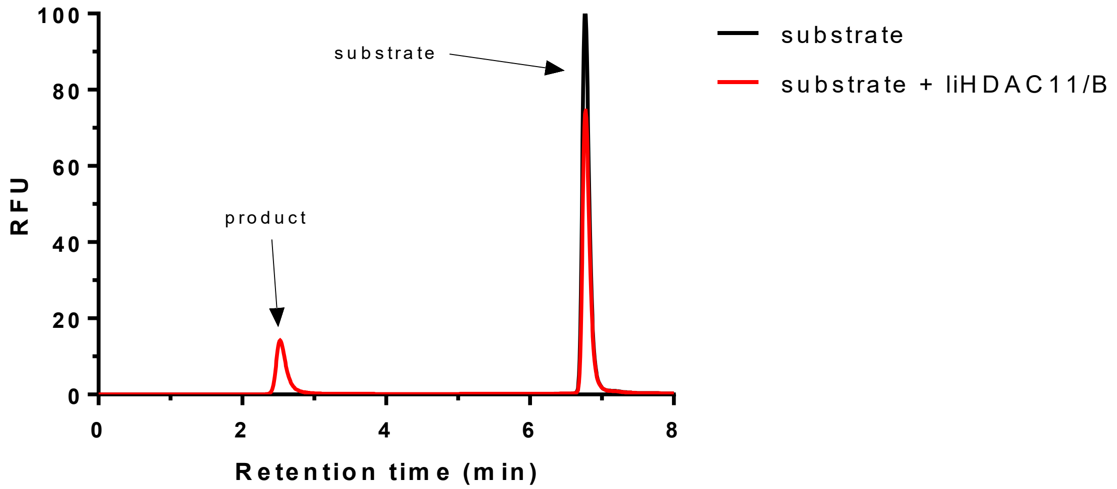

Figure S8. HPLC chromatogram of monitoring HDAC11 enzymatic activity. Example of substrate elution profile. The peptide substrate modified with the octanoyl moiety was incubated with liHDAC11/B (or without the enzyme as a negative control) at 37°C for 30 mins. Decrease in the substrate peak (black) and the concomitant increase in the product concentration (red line) were monitored using HPLC. HDAC11 activity was quantified from integrated product peaks.

Figure S9 (file). Comparative model of cvHDAC11/A generated *in silico* for structural analyses of conserved motifs.

Figure S10 (file). Comparative model of gvHDAC11/A generated *in silico* for structural analyses of conserved motifs.

Figure S11 (file). Comparative model of chrHDAC11-1/A generated *in silico* for structural analyses of conserved motifs.

Figure S12 (file). Comparative model of nvHDAC11/A generated *in silico* for structural analyses of conserved motifs.

Figure S13 (file). Comparative model of lmHDAC11/A generated *in silico* for structural analyses of conserved motifs.

Figure S14 (file). Comparative model of psHDAC11/A generated *in silico* for structural analyses of conserved motifs.

Figure S15 (file). Comparative model of chrHDAC11-2/A generated *in silico* for structural analyses of conserved motifs.

Figure S16 (file). Comparative model of eaHDAC11/A generated *in silico* for structural analyses of conserved motifs.

Figure S17 (file). Comparative model of acibHDAC11/B generated *in silico* for structural analyses of conserved motifs.

Figure S18 (file). Comparative model of liHDAC11/B generated *in silico* for structural analyses of conserved motifs.

Figure S19 (file). Comparative model of cwaHDAC11/B generated *in silico* for structural analyses of conserved motifs.

Figure S20 (file). Comparative model of dbHDAC11/B generated *in silico* for structural analyses of conserved motifs.

Figure S21 (file). Comparative model of pbHDAC11/B generated *in silico* for structural analyses of conserved motifs.

Figure S22 (file). Comparative model of albaHDAC11/B generated *in silico* for structural analyses of conserved motifs.

Figure S23 (file). Comparative model of ttHDAC11/B generated *in silico* for structural analyses of conserved motifs.

Figure S24 (file). Comparative model of chrHDAC11/B generated *in silico* for structural analyses of conserved motifs.

Figure S25 (file). Comparative model of ceHDAC11/B generated *in silico* for structural analyses of conserved motifs.

Figure S26 (file). Comparative model of atHDAC11/B generated *in silico* for structural analyses of conserved motifs.

Figure S27 (file). Comparative model of lmHDAC11/B generated *in silico* for structural analyses of conserved motifs.

Figure S28 (file). Comparative model of dmHDAC11/B generated *in silico* for structural analyses of conserved motifs.

Figure S29 (file). Comparative model of agHDAC11/B generated *in silico* for structural analyses of conserved motifs.

Figure S30 (file). Comparative model of nvHDAC11/B generated *in silico* for structural analyses of conserved motifs.

Figure S31 (file). Comparative model of hsHDAC11/B generated *in silico* for structural analyses of conserved motifs.

Figure S32 (file). Comparative model of mmHDAC11/B generated *in silico* for structural analyses of conserved motifs.

Figure S33 (file). Comparative model of ssHDAC11/B generated *in silico* for structural analyses of conserved motifs.

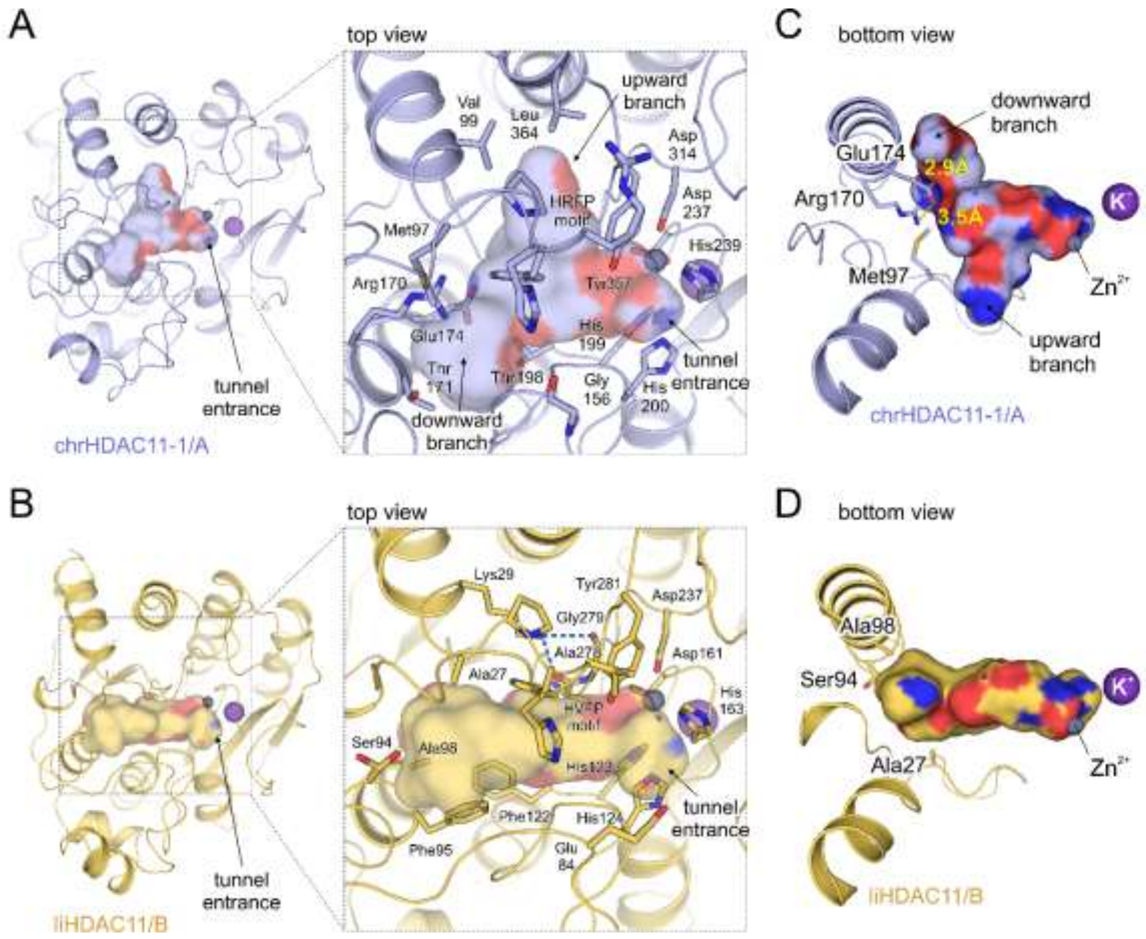

Figure S34. The internal pocket of representative clade A and B enzymes. Panels A, B: Cartoon representation of the crystal structure of chrHDAC11-1/A **(A)** and liHDAC11/B **(B)** with their internal pocket shown as semitransparent surface. Close-up top view shows amino acids shaping the pocket as sticks colored by elements. Panels C, D: Bottom view of the internal pocket of chrHDAC11-1/A **(C)** and liHDAC11/B **(D)** showing the difference in the pocket shape and amino acids at the bottom of the foot pocket. In chrHDAC11-1/A the pocket is shortened by the presence of the Arg170 – Glu174 salt bridge (replacing Ser94 and Ala98 in) with interatomic distances of 2.9 Å and 3.5 Å as well as by substitution of Ala27 (liHDAC1/B) by bulky Met97 in chrHDAC11-1/A. Consequently, the long fatty acids chains cannot be accommodated in the shorter pocket of chrHDAC11-1/A (and clade A enzymes in extenso).

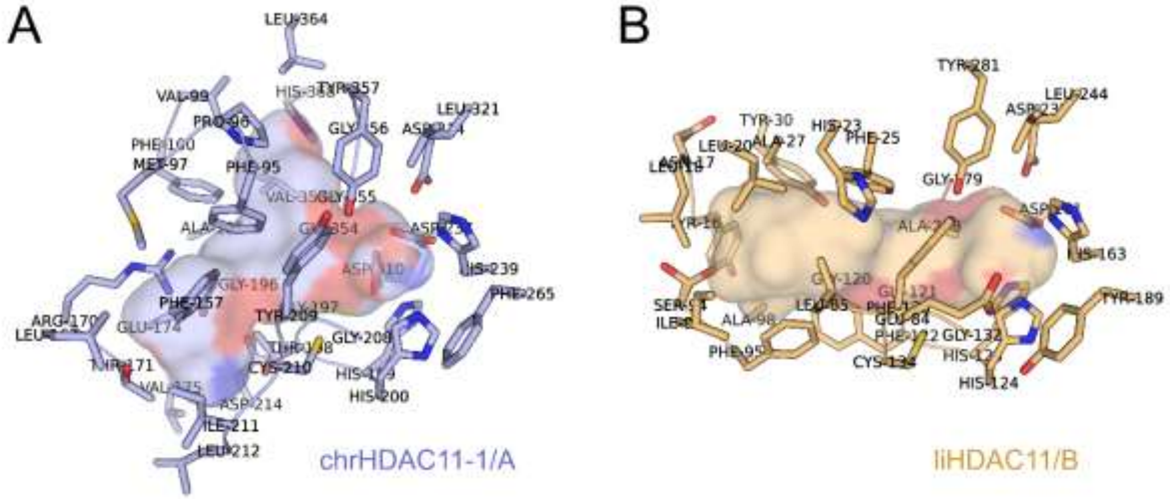

Figure S35. Visualization of the foot pocket of chrHDAC11-1/A and liHDAC11/B. (A) Surface representation of the chrHDAC11-1/A and **(B)** liHDAC11/B foot pockets with amino acids shaping the wall of the pocket shown in stick representation.

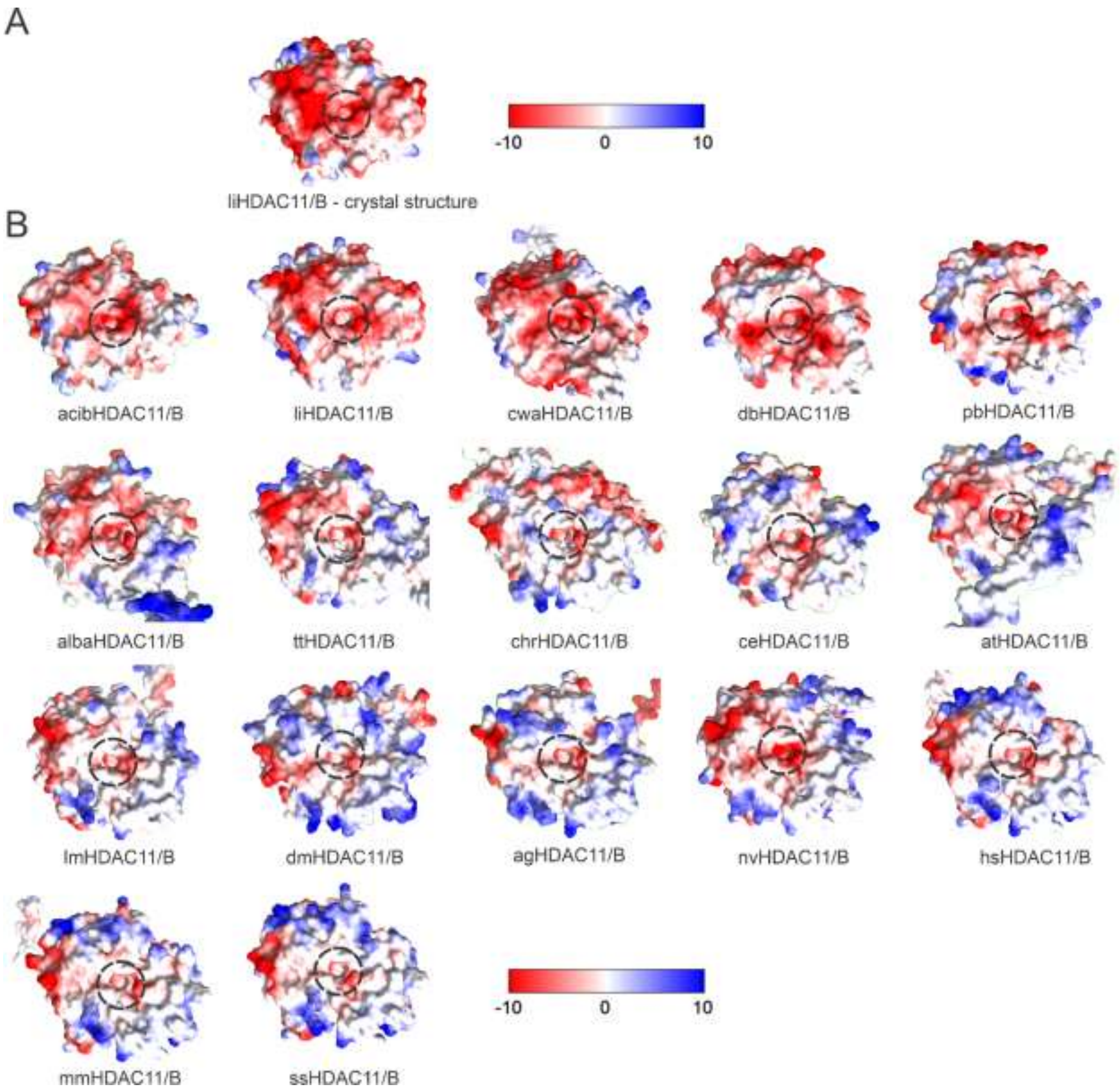

Figure S36. Electrostatic potential of clade B HDACs. (A) X-ray structure of liHDAC11/B. **(B)** AlphaFold models of individual HDAC11 representatives. Clade B enzymes are shown in surface representation colored by electrostatic potential. Electronegative regions are shown in red, electropositive regions in blue, and neutral regions in white. The black circle marks the entrance to the tunnel leading to the active site. The figure was generated using ChimeraX version 1.6.1.0 (Goddard, et al. 2018; Pettersen, et al. 2021; Meng, et al. 2023).

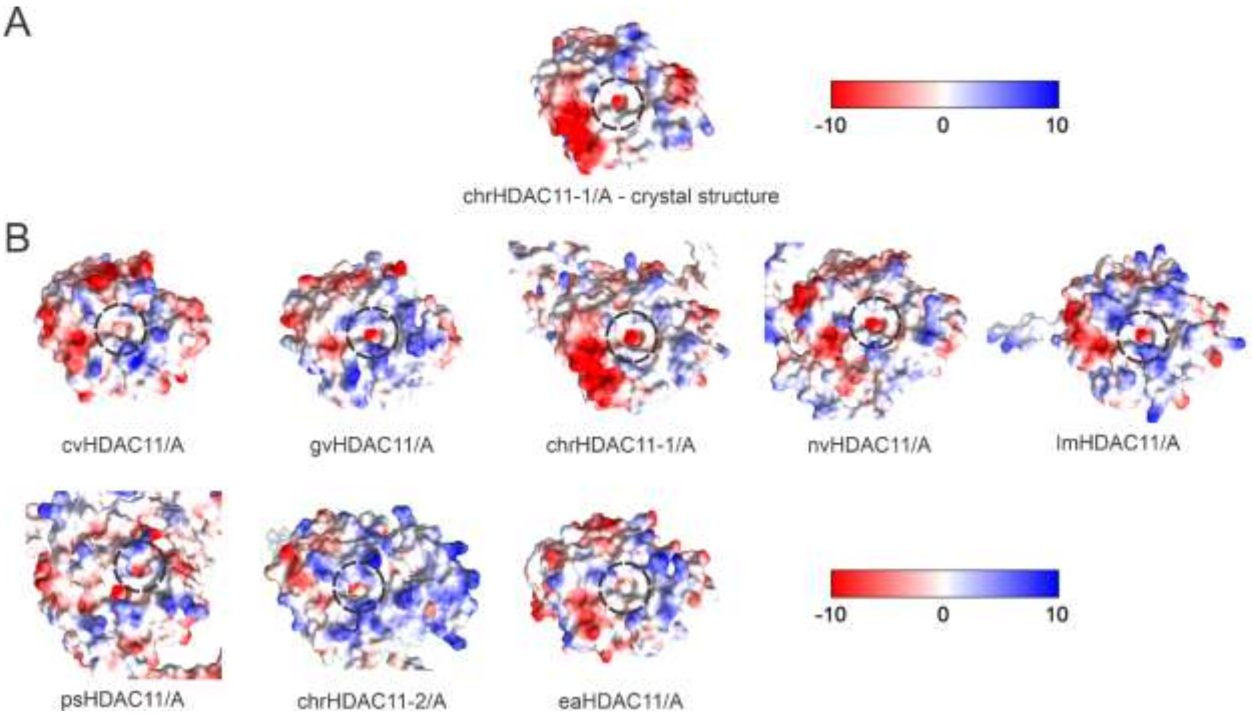

Figure S37. Electrostatic potential of clade A HDACs. **(A)** X-ray structure of chrHDAC11-1/A. **(B)** AlphaFold models of individual HDAC11 representatives. Clade A enzymes are shown in surface representation colored by electrostatic potential. Electronegative regions are shown in red, electropositive regions in blue, and neutral regions in white. The black circle marks the entrance to the tunnel leading to the active site. The figure was generated using ChimeraX version 1.6.1.0.

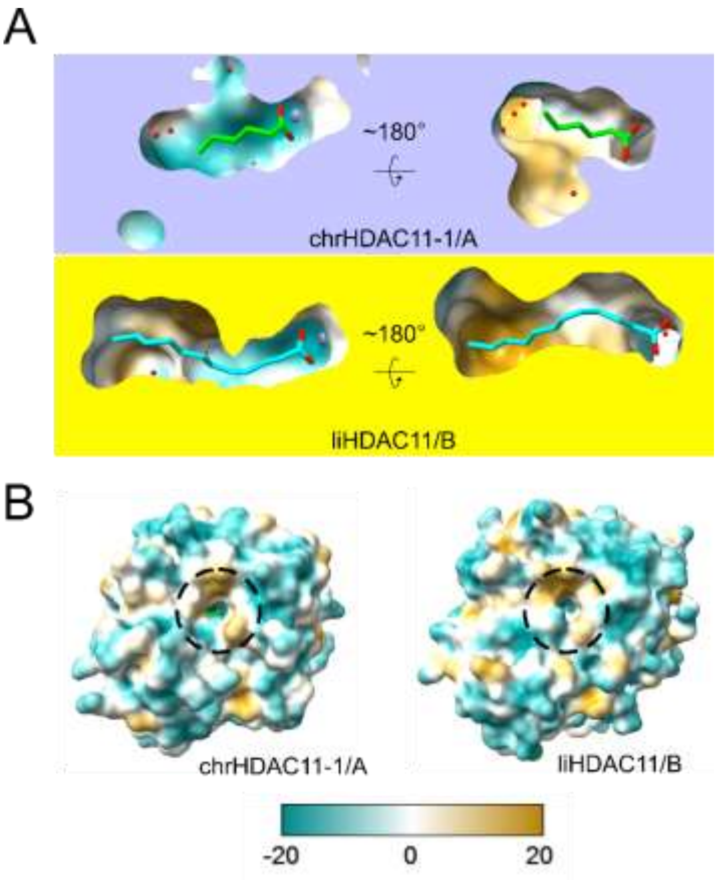

Figure S38. Hydrophobicity analysis of surface and the internal pockets of representative clade A and B enzymes. (A) Hydrophobic surface representation of the internal pocket of chrHD11-1/A and liHD11/B chain A. The tunnel is mostly hydrophilic along one wall (cyan), while mostly hydrophobic (yellow) along the opposite wall. **(B)** Surface representations of chrHD11-1/A and liHD11/B X-ray structures colored by hydrophobicity. Hydrophobic regions are shown in yellow, hydrophilic regions in cyan. The tunnel entrance regions display similar hydrophobic surface characteristics in both structures. The figure was generated using ChimeraX version 1.6.1.0.

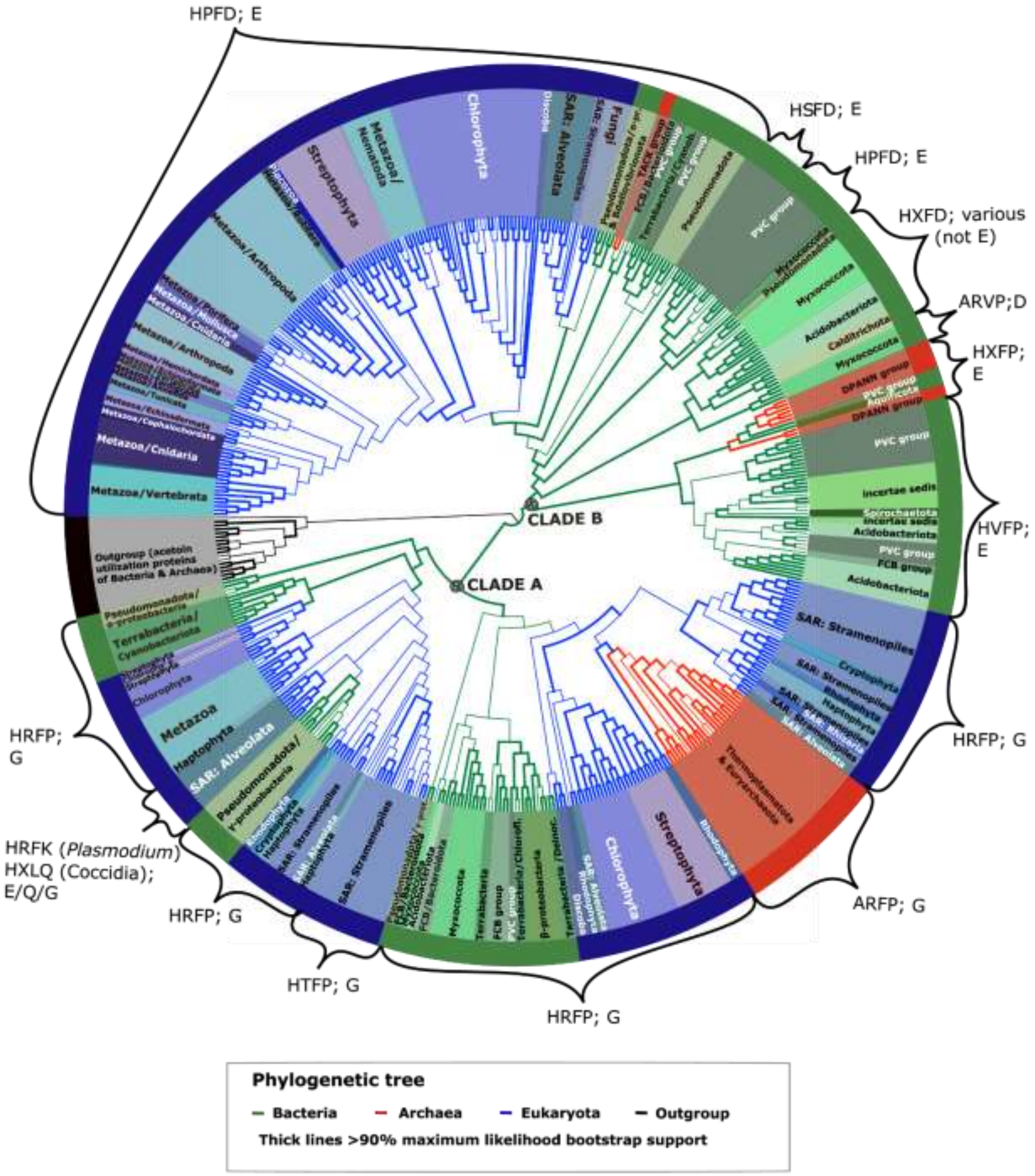

Figure S39. Circle-formatted maximum likelihood phylogenetic tree of 480 class IV HDACs. Variations of the HPFD motif and the amino acid residue in L2 loop corresponding to E94 in hsHDAC11/B are shown for representatives of individual subclades of the A and B clades.

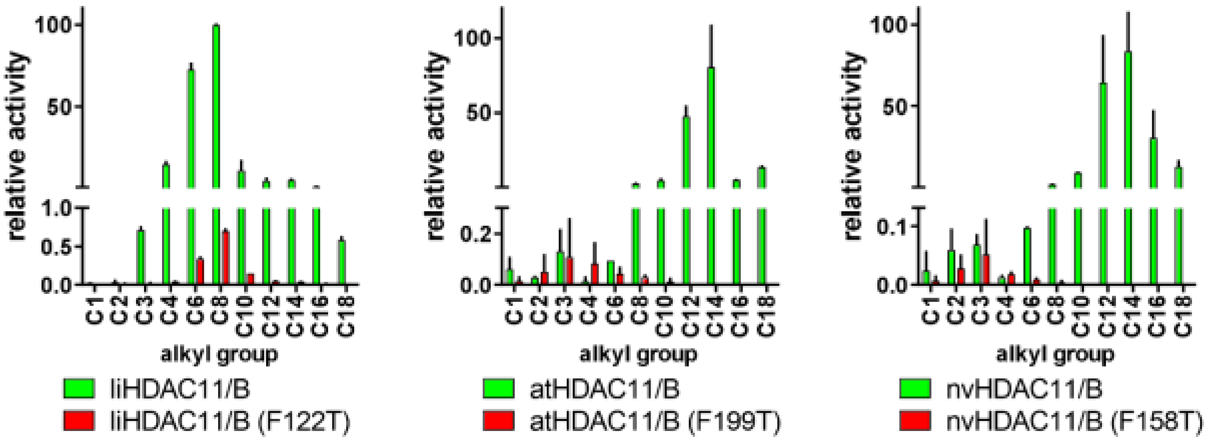

Figure S40. Site-directed mutagenesis of the foot pocket gatekeeper residues. The gatekeeper phenylalanine residues of selected clade B enzymes were mutated to threonine (the clade A signature) and deacylation activities of the mutants compared to the wild-type enzymes. The mutations led to marked decrease in enzymatic activities revealing that the foot pocket gatekeeper is one of the critical signature motifs defining the catalytic activity of clade A vs clade B enzymes.

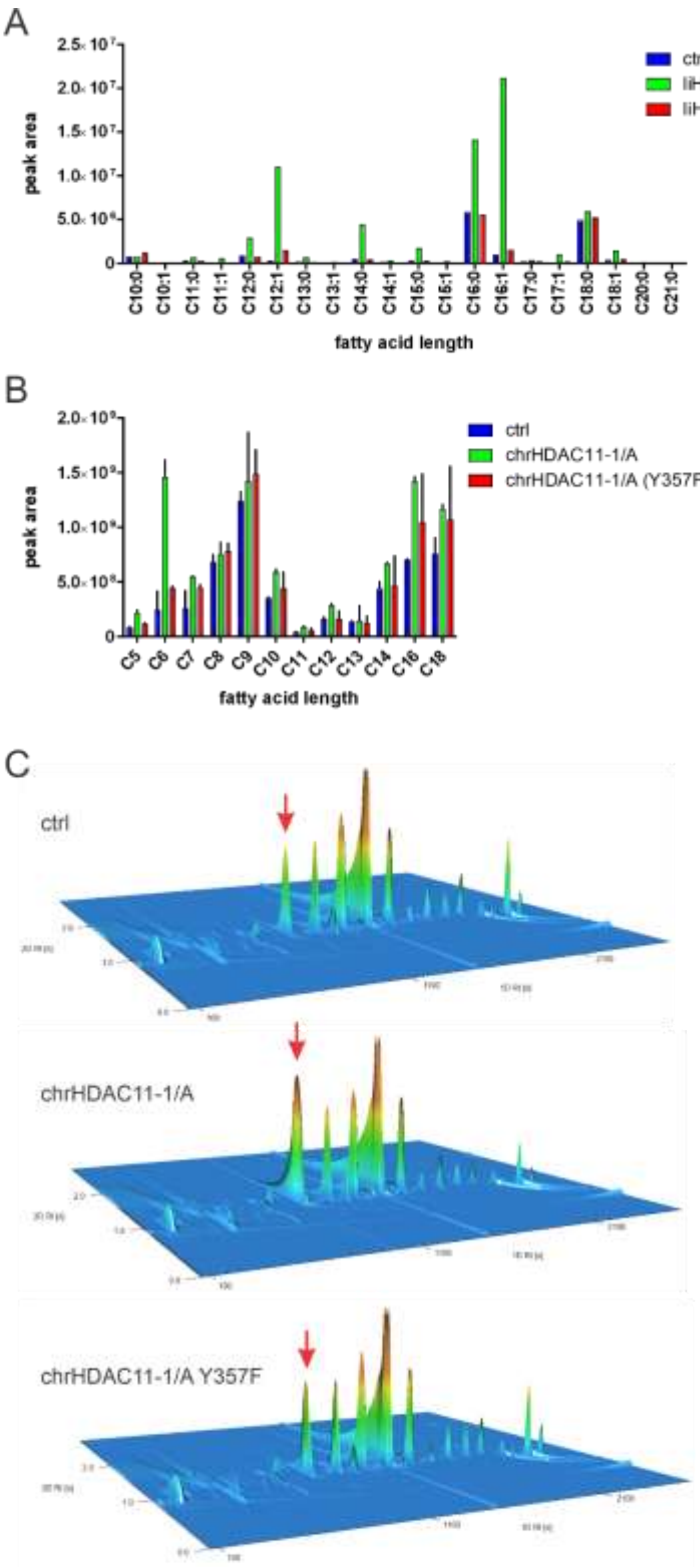

Figure S41. **Identification of fatty acids present in HDAC11 preparations. (A)** Fatty acids identified in liHDAC11/B preparations using LC-MS analysis. Species of the length equal to C12 and C16 were detected prominently in liHDAC11/B when compared to control (His-MBP) and enzymatically inactive variant liHDAC11/B (Y281F). **(B, C)** Identification of foot pocket-resident ligand in chrHDAC11-1/A preparation using GCxGC-MS. (B) Averaged peak areas shown as mean +/- S.D. (n=2). (**C**) 3D visualization of raw data from a representative experiment.

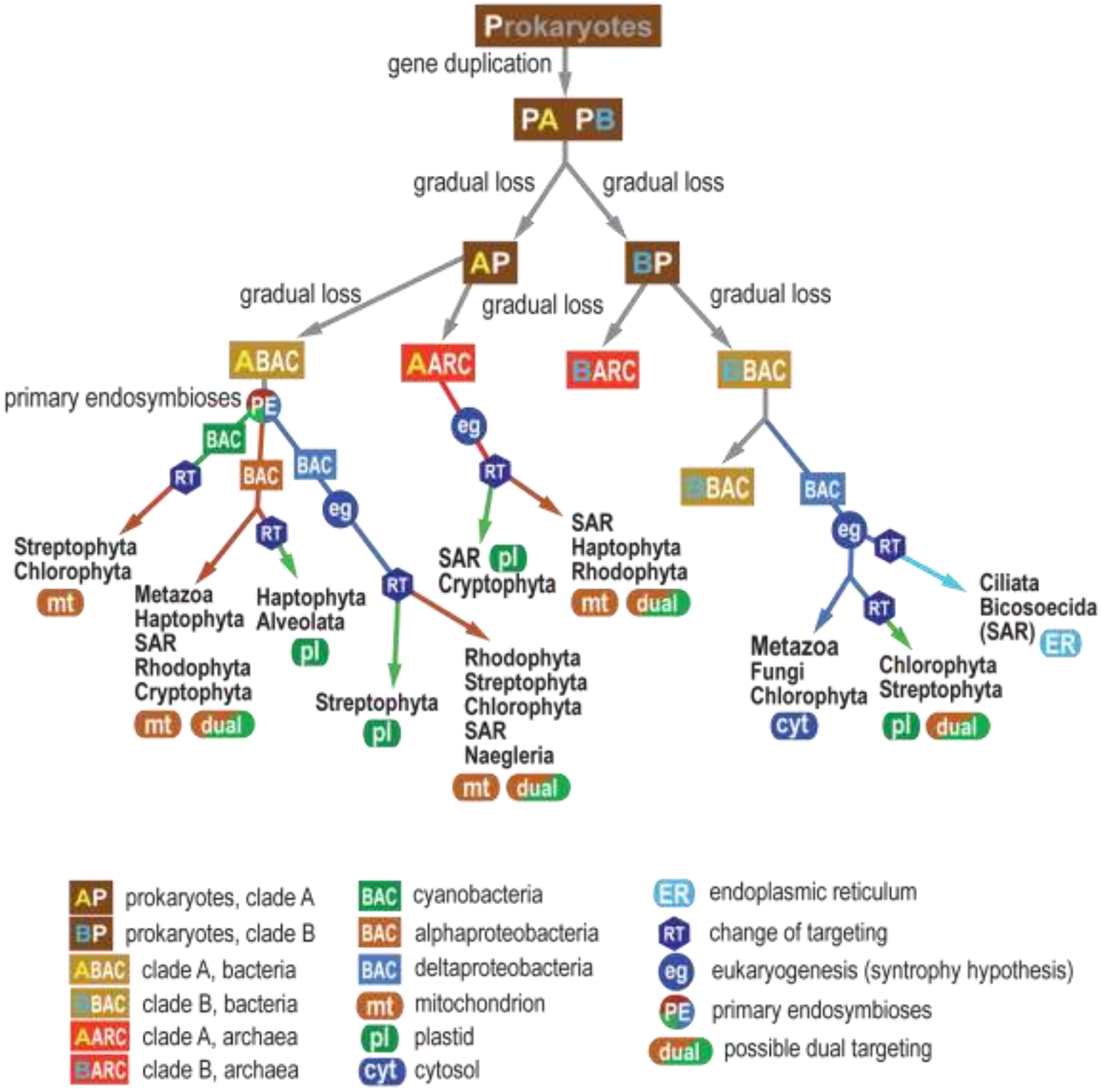

Figure S42. Hypothetical evolution of class IV HDACs.

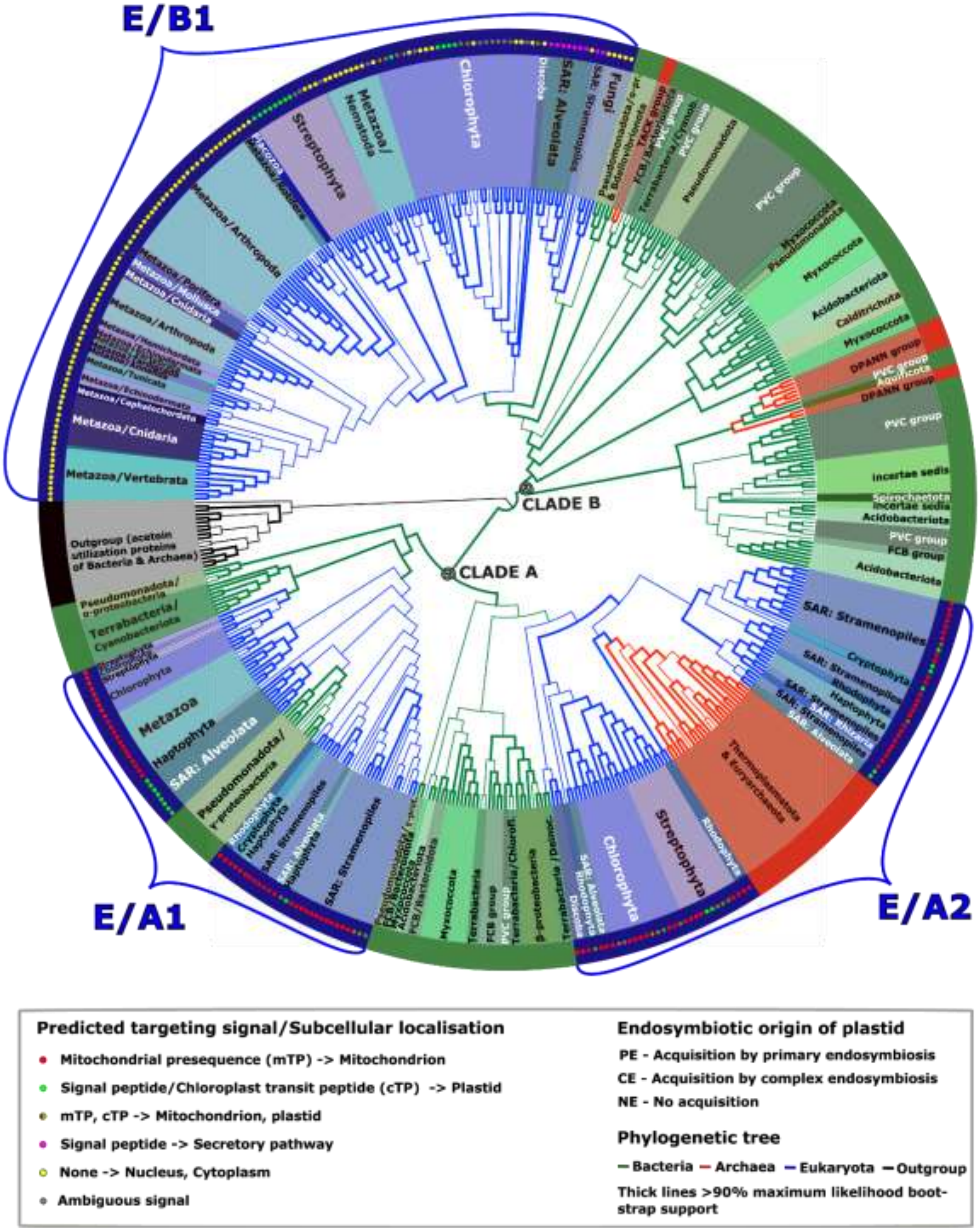

Figure S43. The proposed evolutionary scenario of class IV superimposed on a phylogenetic tree. The four major HDAC clades (ABAC, AARC, BBAC, BARC) with mechanisms of origin detailed in figure S42.

Supplementary Material and Methods

Sample Preparation and 4-I-AMPP⁺ Derivatization

Fatty acids were extracted from the samples following the procedure described in *Sample Preparation for GC-MS – Diazomethane Derivatization*, modified for smaller sample volumes. Briefly, 273 µL of sample containing 1.8 mg of protein was digested with trypsin. Subsequently, 0.10 mL of Milli-Q water containing 56 mg of NaCl and 1 µL of HCl (36%) was added. The mixture was vortexed and extracted twice with 0.50 mL of methyl tert-butyl ether (MTBE) and once with 0.50 mL of a methanol:chloroform mixture (2:1, v/v). The combined organic layers were collected, and the solvents were gently evaporated under a stream of argon.

The extracted fatty acids were derivatized using trifluoroacetic acid salt of 1-(3-(aminomethyl)-4-iodophenyl)pyridin-1-ium (*4-I-AMPP⁺)* according slightly modified method (Narreddula, et al. 2019). The reagent *4-I-AMPP⁺* was kindly provided by Prof. Stephen J. Blanksby (Queensland University of Technology, Australia). The dried extracts were re-dissolved in 25 µL of acetonitrile:dimethylformamide (ACN:DMF, 4:1, v/v) in glass vials. After brief vortexing, 25 µL of 1-hydroxybenzotriazole hydrate (25 mM solution in ACN:DMF, 4:1) and 25 µL of 1-(3-dimethylaminopropyl)-3-ethylcarbodiimide hydrochloride (24 mM solution in ACN:DMF:H_2O, 8:2:1) were added. The reaction mixture was vortexed for 1 min and left at room temperature for 10 min, followed by the addition of 17 µL of the trifluoroacetic acid salt of *4-I-AMPP⁺* solution (40 mM solution in ACN:DMF, 4:1). The resulting mixture was heated at 65 °C for 90 min. After derivatization, 0.30 mL of saturated NaCl solution was added, and the reaction products were extracted twice with 30 µL of MTBE. The combined organic layers were evaporated under argon, and the dried *4-I-AMPP⁺* derivatives were reconstituted in 50 µL of methanol before UHPLC–MS analysis.

UHPLC–MS Analysis of 4-I-AMPP⁺ Fatty Acid Derivatives

Analysis of *4-I-AMPP⁺* fatty acid derivatives was performed using a Vanquish UHPLC system coupled with a TriPlus RTC autosampler and an Orbitrap IQ-X Tribrid mass spectrometer (Thermo Fisher Scientific, MA, USA) equipped with a heated electrospray ionization (HESI) source. Mobile phase A consisted of 0.1% formic acid in water, and mobile phase B was 0.1% formic acid in acetonitrile. Chromatographic separation was achieved on a Waters Acquity UPLC BEH C18 column (2.1 × 50 mm, 1.7 µm) maintained at 45 °C, with a flow rate of 150 µL min^{-1}. The gradient elution program was as follows: 0–1.0 min, 30–30% B; 1.0–3.0 min, 30–65% B; 3.0–15.0 min, 65–70% B; 15.0–22.0 min, 70–80% B; 22.0–28.2 min, 80–100% B; 28.2–29.9 min, 100% B; 29.9–30.0 min, 100–30% B; and 30.0–35.0 min, 30% B. The injection volume was 5 µL. Full-scan and MS/MS spectra were acquired in data-dependent acquisition (DDA) mode under positive ionization polarity over the *m/z* range 250–800, employing ultraviolet photodissociation (UVPD) with calibrated, molecular-weight-dependent activation times. The resolving power of the Orbitrap analyzer was set to 120,000 (at *m/z* 200). The electrospray voltage was 3.6 kV, the capillary temperature 320 °C, sheath gas 35, auxiliary gas 8, and sweep gas 0.5 (arbitrary units).

Data analysis was performed using Xcalibur Qual Browser (Thermo Scientific) and MZmine 4 (Schmid, et al. 2023). Quantitative comparisons of fatty acids were carried out based on the peak areas of reconstructed extracted-ion chromatograms of the corresponding molecular ions.

Supplementary References

Goddard TD, Huang CC, Meng EC, Pettersen EF, Couch GS, Morris JH, Ferrin TE. 2018. UCSF ChimeraX: Meeting modern challenges in visualization and analysis. Protein Sci 27:14–25.

Gore S, García ES, Hendrickx PMS, Gutmanas A, Westbrook JD, Yang HW, Feng ZK, Baskaran K, Berrisford JM, Hudson BP, et al. 2017. Validation of Structures in the Protein Data Bank. Structure 25:1916–1927.

Meng EC, Goddard TD, Pettersen EF, Couch GS, Pearson ZJ, Morris JH, Ferrin TE. 2023. UCSF ChimeraX: Tools for structure building and analysis. Protein Sci 32:e4792.

Narreddula VR, Boase NR, Ailuri R, Marshall DL, Poad BLJ, Kelso MJ, Trevitt AJ, Mitchell TW, Blanksby SJ. 2019. Introduction of a Fixed-Charge, Photolabile Derivative for Enhanced Structural Elucidation of Fatty Acids. Anal Chem 91:9901–9909.

Pettersen EF, Goddard TD, Huang CC, Meng EC, Couch GS, Croll TI, Morris JH, Ferrin TE. 2021. UCSF ChimeraX: Structure visualization for researchers, educators, and developers. Protein Sci 30:70–82.

Schmid R, Heuckeroth S, Korf A, Smirnov A, Myers O, Dyrlund TS, Bushuiev R, Murray KJ, Hoffmann N, Lu M, et al. 2023. Integrative analysis of multimodal mass spectrometry data in MZmine 3. Nature Biotechnology 41:447–449.
