# Supplementary figures and images for: Functional divergence and structural changes of Class IV histone deacetylases (HDACs) across the tree of life
Source: Mol Biol Evol. 2026 Jun 17;43(7):msag150. doi: 10.1093/molbev/msag150 (PMC13325672; doi:10.1093/molbev/msag150)

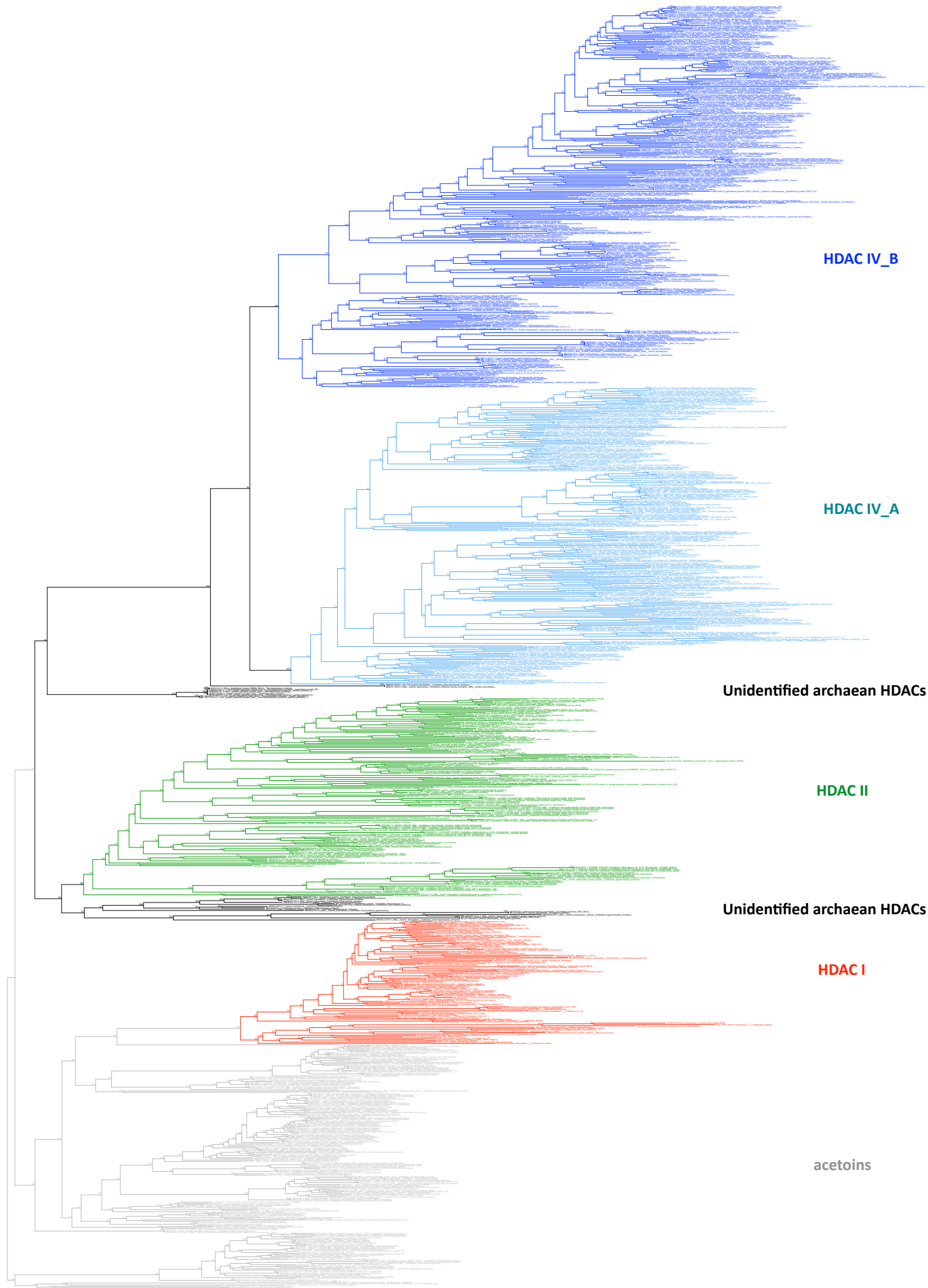

Supplement: msag150_Supplementary_Data [file msag150_supplementary_data.zip › Figure S2.pdf]

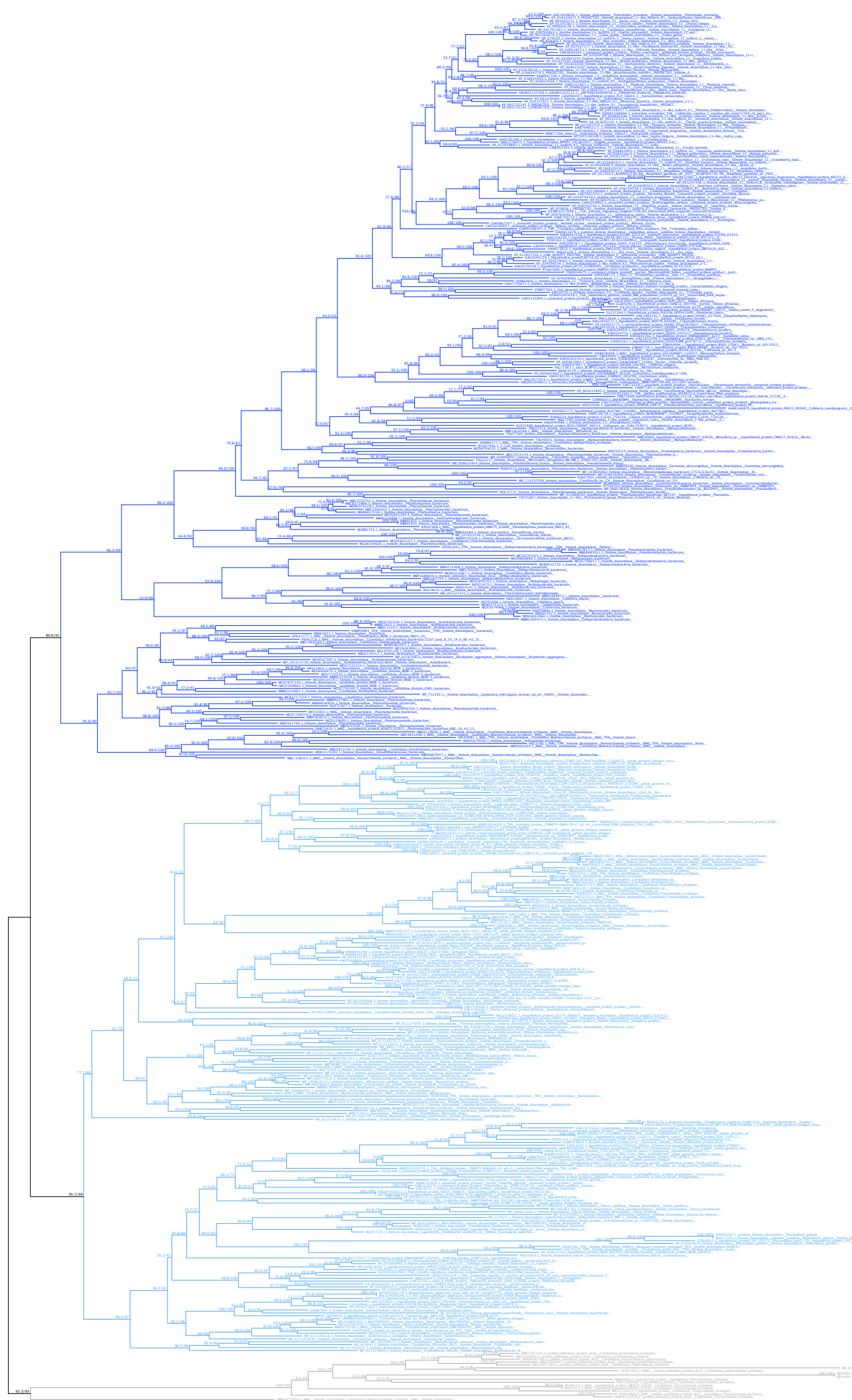

HDAC IV\_B

HDAC IV\_A

acetoin

Supplement: msag150_Supplementary_Data [file msag150_supplementary_data.zip › Figure S3.pdf]
